# Predictive Modeling of Surface Integrity and Material Removal Rate in Computer Numerical Control Machining: Effects of Thermal Conductivity and Hardness

**DOI:** 10.3390/ma18071557

**Published:** 2025-03-29

**Authors:** Mohammad S. Alsoufi, Saleh A. Bawazeer

**Affiliations:** Department of Mechanical Engineering, College of Engineering and Architecture, Umm Al-Qura University, Makkah 21955, Saudi Arabia; mssoufi@uqu.edu.sa

**Keywords:** CNC turning, thermal conductivity, material removal rate (MRR), predictive modeling, surface roughness (R_a_), surface waviness (W_a_), hardness

## Abstract

This study investigates the influence of thermal conductivity and hardness on computer numerical control (CNC) turning performance, focusing on key machining metrics—material removal rate (MRR), surface roughness (*R*_a_), and surface waviness (*W*_a_)—across five engineering materials: aluminum 6061, brass C26000, bronze C51000, carbon steel 1020, and stainless steel 304. Experimental results reveal a strong correlation between material properties and machining efficiency. Materials with high thermal conductivity (>100 W/m·K) exhibited up to 38% higher MRR and improved surface integrity compared to low-conductivity counterparts. Aluminum 6061 achieved the highest MRR (7.5 mm^3^/min at a 0.25 mm/rev feed rate), with the lowest *R*_a_ (~0.58 µm) and *W*_a_ (~0.4576 µm), confirming its excellent machinability and heat dissipation. Conversely, stainless steel 304, characterized by low thermal conductivity (16 W/m·K) and high hardness (210 HBW), recorded the lowest MRR (1.125 mm^3^/min), elevated *R*_a_ (>1.0 µm), and substantial waviness (*W*_a_ ~0.9442 µm), indicating severe tool wear and thermal deformation. A multivariable regression model incorporating cutting speed, feed rate, thermal conductivity, and hardness was developed to predict MRR, achieving high predictive accuracy (*R*^2^ > 0.92) for high-conductivity materials. Deviations of ±0.5 mm^3^/min were observed in harder, low-conductivity materials due to nonlinear effects such as strain hardening and thermal expansion. Measurement uncertainty analysis, with an estimated expanded uncertainty of ±2.5% for MRR and ±0.02 µm for surface metrics, ensures the reliability of these findings. These results underscore the importance of material-specific machining parameter optimization to enhance productivity, surface quality, and tool longevity in high-precision industries, including aerospace, automotive, and biomedical manufacturing.

## 1. Introduction

Computer numerical control (CNC) machining has become a fundamental process in modern manufacturing, offering high precision, repeatability, and surface integrity for critical applications such as aerospace, automotive, biomedical, and high-performance engineering applications. Among the various factors influencing machining efficiency, material removal rate (MRR), surface roughness (*R*_a_), and surface waviness (*W*_a_) play a crucial role in determining the functional and mechanical properties of machined components. These parameters are significantly affected by cutting speed, feed rate, tool geometry, and, most importantly, material properties such as thermal conductivity and hardness [1]. While extensive research has explored the effects of machining parameters on surface quality, limited studies have systematically correlated material thermal conductivity and hardness with CNC turning performance across multiple material types. This lack of comparative analysis presents a challenge in generalizing machining behaviors and optimizing process conditions for different material classes.

Thermal conductivity is a critical factor in machining performance, as it governs the dissipation of heat generated at the tool–workpiece interface. Materials with high thermal conductivity, such as aluminum 6061, enable efficient heat transfer, reducing tool wear and improving surface finish [2]. In contrast, low-thermal-conductivity materials, such as stainless steel 304, accumulate heat in the cutting zone, leading to increased tool degradation, higher cutting forces, and potential surface defects [3]. Similarly, material hardness affects machining efficiency, with harder materials exhibiting greater cutting resistance, resulting in higher tool wear rates, increased surface roughness, and greater surface waviness [4]. The interplay between these two properties influences MRR and surface texture and the overall economic and operational efficiency of CNC turning processes.

Despite advancements in machining research, previous studies have primarily focused on single-material investigations, making it difficult to extrapolate results across a diverse range of engineering materials. Furthermore, while much research has examined surface roughness, the effects of machining conditions on surface waviness remain largely underexplored despite its significant impact on functional performance, fatigue resistance, and component longevity [5]. This study aims to fill these gaps by conducting a comprehensive experimental evaluation of five engineering materials—aluminum 6061, brass C26000, bronze C51000, carbon steel 1020, and stainless steel 304—analyzing their machining performance under identical CNC turning conditions. A predictive model will also be developed to establish quantitative relationships between material properties, machining parameters, and surface integrity outcomes, thereby providing data-driven insights for optimizing CNC machining efficiency.

## 2. Literature Review

### 2.1. Surface Integrity in CNC Machining

Surface integrity is a key determinant of component performance, affecting machined parts’ wear resistance, fatigue strength, and adhesion properties [6]. It also significantly influences wettability, as surface roughness and waviness affect liquid spreading, lubrication, and coating behavior—factors particularly relevant in tribological and biomedical applications [7]. Surface roughness (*R*_a_) measures micro-scale variations, while surface waviness (*W*_a_) reflects larger-scale deviations caused by machine vibrations, tool deflection, and thermal effects [8]. Aurich et al. emphasized that surface texture plays a critical role in functional performance, with both surface roughness and surface waviness contributing to overall contact mechanics and tribological properties [9]. While surface roughness has been widely studied, surface waviness has received considerably less attention despite its importance in dynamic loading conditions and high-precision applications [10].

Studies have shown that higher feed rates tend to increase surface roughness, while higher cutting speeds improve surface finish by reducing built-up edge formation and tool-chip adhesion effects [11]. However, excessive cutting speeds can introduce thermal damage, negatively affecting surface integrity and residual stress distributions [12]. Additionally, the depth of cut significantly influences surface waviness, where deeper cuts lead to increased tool deflection and non-uniform surface deviations [13]. These findings highlight the complex interaction between machining parameters and material properties, necessitating a comprehensive approach to optimize CNC turning performance.

### 2.2. Influence of Material Properties on Machining Performance

Materials’ mechanical and thermal properties directly influence their machinability, affecting tool wear, chip formation mechanisms, and final surface integrity [14]. Günay and Korkmaz demonstrated that materials with high hardness exhibit greater cutting resistance, leading to higher tool wear and increased roughness values [15]. Debnath et al. examined the effects of thermal conductivity on surface finish in aerospace alloys, confirming that low-thermal-conductivity materials experience localized heat accumulation, which accelerates tool degradation and reduces surface quality [16].

Aluminum 6061, with a high thermal conductivity of 205 W/m·K and a moderate hardness of 95 HBW, typically results in smooth surfaces and high MRR, making it an ideal material for high-speed manufacturing applications [17]. Brass C26000 and bronze C51000 offer moderate machinability, balancing thermal conductivity and hardness effects to achieve acceptable roughness and waviness levels. However, carbon steel 1020 and stainless steel 304 present greater machining challenges due to higher hardness and lower thermal conductivity, leading to higher cutting forces, increased tool wear, and deteriorated surface integrity [18].

The influence of these material properties on MRR and surface texture needs further exploration to establish generalized machining guidelines applicable across diverse material classes. In particular, recent studies have clarified thermal conductivity’s role in shaping surface integrity during machining. Günay and Korkmaz [15] confirmed that materials with low thermal conductivity exhibit increased heat accumulation in the cutting zone, leading to tool wear acceleration and surface finish deterioration. Pawanr and Gupta [6] further emphasized that poor thermal dissipation promotes localized thermal deformation and increases surface waviness, particularly in stainless steels. Isakson et al. [19] also demonstrated that thermal conductivity plays a pivotal role in shaping surface topography and influencing residual stress distributions, especially under high-speed machining conditions. These findings underline the importance of considering thermal transport properties when evaluating surface quality outcomes in CNC turning operations.

### 2.3. Predictive Modeling for Machining Performance

Accurate predictive modeling is essential for optimizing machining parameters and reducing process inefficiencies. Several approaches have been proposed to model MRR, surface roughness, and tool wear, ranging from empirical regression models to machine learning techniques [20]. Patel et al. developed a linear regression model to predict surface roughness based on cutting speed, feed rate, and tool geometry, achieving a high correlation between machining inputs and roughness outcomes [21]. However, such models often struggle to capture nonlinear effects, such as strain hardening, thermal expansion, and tool wear progression, particularly in hard-to-machine materials [19].

Machine-learning-based models, such as artificial neural networks (ANN) and support vector machines (SVM), have demonstrated superior predictive capabilities for complex machining processes [22]. These models can integrate material properties, cutting conditions, and real-time sensor feedback to enhance accuracy in predicting MRR, roughness, and waviness evolution. However, the application of such models remains limited in multi-material studies, and their effectiveness in capturing variations across different thermal and mechanical properties requires further validation [23].

### 2.4. Research Gap and Objectives

Despite significant advancements in machining research, there remains a notable gap in studies that comprehensively analyze the combined effects of material properties and machining parameters on both surface roughness and surface waviness, two critical components of surface integrity that directly influence the functional performance, fatigue resistance, and dimensional accuracy of machined components. While material removal rate (MRR) and surface roughness have been extensively investigated, surface waviness remains underexplored despite its significant role in contact mechanics, sealing performance, and high-precision applications [24]. Most previous studies have focused on single-material analyses, which limits the ability to generalize machining trends across diverse engineering alloys with varying thermal and mechanical properties.

This study aims to address these critical gaps through a systematic experimental investigation involving five distinct engineering materials—aluminum 6061, brass C26000, bronze C51000, carbon steel 1020, and stainless steel 304—selected for their wide range of thermal conductivity (16 to 205 W/m·K) and hardness values (95 to 210 HBW). By varying key machining parameters, including cutting speed, feed rate, and depth of cut, the research provides a quantitative assessment of how these factors collectively influence MRR, surface roughness, and waviness. Results indicate that materials with higher thermal conductivity and lower hardness, such as aluminum 6061, achieve superior machinability, evidenced by higher MRR and lower surface irregularities. In contrast, harder, low-conductivity materials like stainless steel 304 exhibit increased tool wear, reduced MRR, and degraded surface integrity.

In addition to the experimental investigation, the study develops predictive models that integrate material properties and machining parameters, achieving high predictive accuracy (*R*^2^ > 0.92) for MRR and significant reliability in forecasting surface quality variations. These models provide valuable insights for optimizing CNC machining processes, enabling improved process planning, tool wear mitigation, and material selection strategies in high-performance manufacturing environments. By systematically addressing the combined effects of thermal and mechanical properties with process parameters, this research significantly contributes to the advancement of precision machining methodologies, promoting enhanced efficiency, product quality, and economic sustainability across various industrial applications.

## 3. Methodology

This section outlines the experimental setup, material selection, material removal rate (MRR) computation, predictive modeling approach, and surface integrity assessment employed in this study. The methodology was structured to systematically analyze the influence of thermal conductivity and hardness on CNC turning performance, focusing on MRR, surface roughness, and surface waviness.

### 3.1. Experimental Setup

The machining experiments were conducted using a high-precision Gate-Eclipse ECL-400 CNC lathe (Gate Machinery International Ltd., Derbyshire, UK), designed to provide exceptional rigidity and machining accuracy for precision manufacturing applications. This advanced lathe was equipped with a Fagor CNC control system, allowing for precise and repeatable adjustments of machining parameters, including cutting speed, feed rate, and depth of cut. The machine’s multi-axis capability ensured consistent cutting operations, while its integrated cooling system helped maintain thermal stability and minimize tool wear, thus enhancing process reliability and extending tool life. All experiments were conducted in a temperature-controlled laboratory environment maintained at 22 ± 1 °C with a relative humidity of 50 ± 5% to eliminate any influence of environmental variations on machining outcomes.

Secure clamping of the workpieces using a high-rigidity three-jaw chuck was used to ensure mechanical stability during the machining process, preventing vibrations and positional shifts that would otherwise compromise machining consistency. All machining trials were performed under dry-cutting conditions to isolate the thermal effects of machining from those introduced by coolant or lubrication. This approach enabled a direct assessment of each material’s inherent heat dissipation characteristics and their corresponding impact on surface integrity and machining performance.

Tungsten carbide cutting inserts conforming to ISO specification CNMG120408-PM [25], coated with titanium aluminum nitride (TiAlN), were employed due to their high wear resistance and suitability for a wide range of materials. To maintain cutting consistency throughout the experiments, inserts were replaced after every five machining passes or when the measured flank wear exceeded 0.3 mm, following the guidelines established in ISO 3685 [26]. Machining parameters were carefully selected to align with the mechanical and thermal properties of the tested materials, ensuring fair and comparable evaluations. Five cutting speeds (30, 30, 60, 90, and 120 m/min) and five feed rates (0.05, 0.10, 0.15, 0.20, and 0.25 mm/rev for aluminum alloy 6061 (SpecLine Arabia Company Ltd., Al-Jubail, Saudi Arabia) and bronze C51000 (Al Mokahal Co. Location, Al-Jubail, Saudi Arabia); and 0.025, 0.05, 0.075, 0.100, and 0.125 for brass C26000, carbon steel 1020, and stainless steel 304) were utilized, with the depth of the cut maintained at a constant value of 0.25 mm. These parameters were chosen to reflect practical industrial machining conditions while allowing for a comprehensive exploration of how varying cutting inputs affect machining outcomes across different materials.

Each machining condition was repeated three times for every material to guarantee statistical reliability. Machining time was automatically recorded through the CNC controller, providing precise timing data for material removal rate (MRR) calculations. Mass loss measurements were conducted using a high-precision digital balance with an accuracy of ±0.001 g, ensuring reliable quantification of material removal. The collected data from the three repetitions under each condition were averaged to mitigate random measurement anomalies and enhance result reliability.

Uncertainty analysis was performed to assess the reliability of the measured data. The main contributors to uncertainty included mass measurement error, CNC timing precision, and variations resulting from tool wear. Applying the propagation of uncertainty method following the Guide to the Expression of Uncertainty in Measurement (GUM) standards, the expanded uncertainty (with a coverage factor *k* = 2) for MRR was estimated to be ±2.5%. This consideration ensures that the experimental results account for measurement and operational variabilities inherent in the machining process.

This experimental setup ensured high reproducibility and robust data collection by meticulously controlling machining parameters, environmental factors, and measurement protocols. The rigor applied to the experimental design provides a solid foundation for the subsequent evaluation of material removal efficiency, thermal effects, and surface integrity in CNC turning operations, ensuring that the study’s conclusions are statistically sound and industrially relevant.

Although direct thermal imaging was not employed in this study, thermal effects were inferred from comparative performance metrics, such as MRR and surface integrity, across materials with distinct thermal conductivities under identical machining conditions.

### 3.2. Material Tested

Five engineering materials were selected to investigate the influence of thermal conductivity and hardness on CNC turning performance. These materials exhibited distinct machinability characteristics, covering a broad spectrum of thermal and mechanical properties, which allowed for a comprehensive evaluation of their behavior under identical machining conditions. The selection aimed to analyze how heat dissipation (thermal conductivity) and cutting resistance (hardness) affect material removal rate (MRR) and surface integrity.

Table 1 presents the selected materials’ thermal conductivity, hardness, and machinability index. Aluminum 6061, with the highest thermal conductivity (205 W/m·K) and relatively low hardness (95 HBW), was classified as highly machinable, making it an ideal material for high-speed manufacturing applications. Brass C26000 and bronze C51000, with thermal conductivities of 109 W/m·K and 60 W/m·K, respectively, exhibited moderate hardness values (115–112 HBW) and were categorized as medium-machinability materials.

In contrast, carbon steel 1020 and stainless steel 304 represented low- and very-low-machinability materials, respectively. Carbon steel 1020, with a thermal conductivity of 51 W/m·K and a hardness of 150 HBW, demonstrated higher cutting resistance, which could impact tool wear and surface roughness. Stainless steel 304, with the lowest thermal conductivity (16 W/m·K) and highest hardness (210 HBW), was the most challenging material to machine, as it was expected to exhibit higher cutting forces, increased tool wear, and poor heat dissipation, leading to potential surface quality issues.

These materials were chosen to ensure a diverse representation of machining behaviors related to heat dissipation and mechanical hardness, providing valuable insights into optimizing machining parameters for different material classes. This selection also allowed for a systematic study of material removal rate (MRR) and surface integrity variations across a wide range of machining conditions.

### 3.3. MRR Computation

The material removal rate (MRR) was computed to quantitatively evaluate the machining efficiency of each material under varying cutting conditions. The mass loss method was employed to ensure high accuracy and reliability, which effectively accounts for factors such as material deformation and chip fragmentation, which are often overlooked in conventional volumetric approaches. The MRR calculation was based on the following equation:

(1) $$MRR\;=\;\frac{∆W}{\varrho \;\times \;t}$$

where:

∆W represents material mass loss (g);

ϱ is the material density (g/mm^3^);

t is the machining time (min).

A high-precision digital balance with an accuracy of ±0.001 g was used to measure the mass loss. The machining time was automatically recorded via the CNC control system, ensuring consistent timing accuracy across all trials and eliminating potential human error associated with manual measurements. Material densities were obtained from ASTM standard references [27], with the following values applied: aluminum 6061 (2.70 g/cm^3^), brass C26000 (8.53 g/cm^3^), bronze C51000 (8.82 g/cm^3^), carbon steel 1020 (7.87 g/cm^3^), and stainless steel 304 (7.90 g/cm^3^). These standardized density values ensured uniformity in the MRR calculations and facilitated cross-material comparisons.

Each test condition was repeated thrice per material to enhance statistical reliability. The recorded MRR values from the repeated trials were averaged to mitigate random fluctuations and measurement anomalies arising from tool wear, chip adherence, or environmental factors. This systematic repetition strengthened the robustness of the data, ensuring that the calculated MRR values accurately reflected material-specific machinability trends.

An uncertainty analysis was conducted to quantify potential variations in MRR measurements. Primary contributors to uncertainty included mass measurement errors, CNC-recorded machining time precision, and material density deviations. Using the propagation of uncertainty method in accordance with the Guide to the Expression of Uncertainty in Measurement (GUM) [28], the expanded uncertainty (with a coverage factor *k* = 2) for the MRR values was calculated to be approximately ±2.5%. This level of uncertainty is considered acceptable for high-precision machining assessments and provides confidence in the reliability of the reported results.

To further explore the influence of machining parameters and material properties on MRR, a comprehensive dataset was compiled, incorporating cutting speed (*V*_c_), feed rate (*f*), thermal conductivity (*λ*), and hardness (HBW) for each material. This dataset served as the foundation for the predictive modeling approach outlined in the subsequent section, enabling a deeper understanding of how material characteristics and process parameters interact to influence machining efficiency.

This section incorporates stringent measurement protocols, quantifies uncertainty, and gathers comprehensive data to establish a reliable foundation for analyzing material removal trends and facilitates the development of precise predictive models for CNC turning operations.

### 3.4. Predictive Model

A multivariable regression model was developed to establish a data-driven framework for predicting MRR, integrating machining parameters and material properties as independent variables. The model was formulated as:

(2) $$MRR\;=\;C_{0}\;+\;C_{1}V_{c}\;+\;C_{2}f\;+\;C_{3}\lambda \;+\;C_{4}HBW\;+\;\varepsilon$$

where:

$C_{0}$—intercept (constant term);

$C_{1},\;C_{2},\;C_{3},\;C_{4}$—regression coefficients corresponding to each independent variable;

ε—error term accounting for random variability;

$V_{c}$—cutting speed (m/min);

f—feed rate (mm/rev);

λ—thermal conductivity (W/m·K);

HBW—Brinell hardness.

The model was trained and validated using experimental MRR data collected from five engineering materials: aluminum alloy 6061, brass C26000, bronze C51000, carbon steel 1020 annealed, and stainless steel 304 annealed. Each material was tested under a matrix of four cutting speeds (30, 60, 90, and 120 m/min) and four feed rates (0.05, 0.10, 0.15, and 0.20 mm/rev), resulting in a comprehensive dataset that ensured accurate and generalizable predictions across various machining conditions.

The model was statistically validated by calculating the coefficient of determination (*R*^2^) and residual diagnostics. The regression analysis yielded a high goodness-of-fit value (*R*^2^ = 0.927), indicating that approximately 92.7% of the variance in MRR could be explained by the selected machining parameters and material properties. This strong correlation emphasizes the significant influence of cutting speed, feed rate, thermal conductivity, and hardness on material removal efficiency.

The model demonstrated high predictive accuracy for materials with higher thermal conductivity, such as aluminum 6061 and brass C26000, with residual errors generally within ±0.3 mm^3^/min. This accuracy is attributed to the linear relationship between thermal conductivity and heat dissipation, which directly impacts chip formation and cutting forces. Conversely, the model exhibited higher residual errors (up to ±0.5 mm^3^/min) for harder materials like stainless steel 304 and carbon steel 1020. These deviations stem from nonlinear machining effects, including strain hardening, thermal expansion, and progressive tool wear, which were not fully captured by the linear regression approach.

The model’s performance shows a close alignment between predicted and experimental values for most materials, with thermally conductive materials exhibiting slight discrepancies. The residual analysis reveals that residuals are more pronounced at higher feed rates and cutting speeds for harder materials, underscoring the influence of unmodeled nonlinear factors.

While the current model provides valuable insights into MRR trends and offers practical predictive capabilities for most engineering materials, the observed deviations for materials with high hardness and low thermal conductivity highlight areas for future improvement. Incorporating advanced modeling techniques, such as nonlinear regression or machine learning algorithms, could enhance predictive accuracy, particularly under complex machining conditions involving work-hardening materials and high cutting forces.

### 3.5. Surface Integrity Integration

Surface integrity was assessed using a high-precision Taly-Surf^®^ profilometer (Taylor Hobson Precision, Inc., Leicester, UK), a nanometer-resolution stylus-based instrument capable of accurately measuring surface roughness (*R*_a_), waviness (*W*_a_), skewness (*R*_sk_, *W*_sk_), and kurtosis (*R*_ku_, *W*_ku_). The device was configured with a 2 µm conisphere stylus, applying a measurement force of 0.7 mN and operating at a traverse speed of 0.5 mm/s, ensuring high-resolution and repeatable surface texture evaluations across all tested materials.

All measurements were conducted in a vibration-isolated room maintained at 20 ± 0.5 °C to eliminate external influences and maintain measurement precision. The stylus tip was examined every 20 measurements for signs of wear to prevent measurement drift and maintain consistent readings, and no noticeable changes were observed during the experimental runs.

Measurement zones were evenly spread along the machined surface at equidistant intervals to ensure complete surface representation. Each material sample was evaluated at five distinct zones, with three independent measurements per zone to account for localized surface anomalies and enhance statistical reliability. The recorded data were averaged to provide a representative surface integrity profile for each material.

Data processing protocols included applying a linear least-squares regression to eliminate baseline drift, ensuring the accuracy of roughness and waviness profiles. A Gaussian low-pass filter, conforming to ISO 4287 standards [29], was employed to separate micro-scale roughness from macro-scale waviness components. This separation was critical to our analysis, as roughness and waviness reflect different spatial scales of surface topography and influence distinct functional aspects—such as tribological performance in the case of roughness and fatigue or sealing behavior in the case of waviness. The measurement uncertainty for *R*_a_ and *W*_a_ was determined to be ±0.02 µm, calculated following ISO 25178 guidelines [30], ensuring that all reported surface integrity metrics fall within acceptable confidence limits.

Integrating surface integrity assessments with material properties (thermal conductivity, hardness) and machining parameters (cutting speed, feed rate) provides a comprehensive understanding of how material characteristics influence surface quality in CNC turning operations. Notably, materials with higher thermal conductivity and lower hardness generally exhibited lower *R*_a_ and *W*_a_ values, while harder, less conductive materials displayed more pronounced surface irregularities.

This correlation between MRR trends and surface integrity outcomes underscores the importance of material-specific machining strategies. By linking MRR to surface quality metrics, this study delivers actionable insights for optimizing CNC machining parameters to balance productivity with desired surface finish quality, which is particularly relevant for high-precision sectors such as aerospace, automotive, and biomedical manufacturing.

### 3.6. Uncertainty and Error Analysis

A comprehensive uncertainty and error analysis was carried out to ensure the experimental findings were accurate and reliable, in accordance with the Guide to the Expression of Uncertainty in Measurement (GUM) standards. This analysis accounted for uncertainties arising from measuring the material removal rate (MRR), surface integrity parameters (surface roughness, *R*_a_, and surface waviness, *W*_a_), and the control of machining parameters.

For the MRR measurements, which were determined using the mass loss method, primary sources of uncertainty included the accuracy of the digital balance (±0.001 g), slight variations in the material density values (±0.5%), and timing precision during the machining process (±0.2 s). The combined standard uncertainty for MRR was calculated by applying standard error propagation methods, resulting in an expanded uncertainty of approximately ±2.5% at a 95% confidence level. This level of precision ensures that the observed differences in MRR across materials and machining conditions are statistically meaningful and not the result of random measurement errors.

Surface integrity measurements were conducted using a Taly-Surf^®^ profilometer with a conisphere stylus and a measurement resolution of ±0.01 µm. Sources of uncertainty in these assessments included the instrument’s inherent resolution limit, stylus positioning repeatability (±0.015 µm), and baseline drift corrected through linear least-squares regression. Combining these factors resulted in an expanded uncertainty of ±0.02 µm for both *R*_a_ and *W*_a_ measurements. These low uncertainty values reflect the high precision and repeatability of the surface texture evaluations, confirming the validity of the recorded variations in surface quality.

Machining parameter settings on the Gate-Eclipse ECL-400 CNC lathe also contributed to the overall uncertainty, albeit to a lesser extent. The cutting speeds exhibited a variation of approximately ±1% due to system calibration tolerances, while feed rates and depth of cut displayed uncertainties of ±0.005 mm/rev and ±0.02 mm, respectively. Despite these small deviations, the controlled experimental environment and consistent use of the same equipment throughout the trials minimized the influence of these factors on the overall results.

The integration of all identified sources of uncertainty confirms that the experimental trends observed in MRR, surface roughness, and waviness are robust and reliable. Notably, the strong statistical correlations identified between thermal conductivity, hardness, and machining outcomes (*R*^2^ > 0.92) remained valid even when accounting for measurement uncertainties. Although residual deviations were more pronounced for harder materials such as stainless steel 304, primarily due to nonlinear effects like strain hardening and tool wear, these variations fell within the calculated uncertainty margins.

In conclusion, the comprehensive uncertainty analysis ensures the credibility of the experimental findings, the accuracy of the predictive models, and the reliability of the conclusions drawn from the data. By maintaining a consistent and controlled methodology, this study offers statistically significant insights into the effects of material properties and machining parameters on CNC turning performance, thereby providing a valuable foundation for future process optimization in precision manufacturing.

## 4. Results and Discussion

### 4.1. Influence of Machining Parameters on MRR

**Figure 1:**
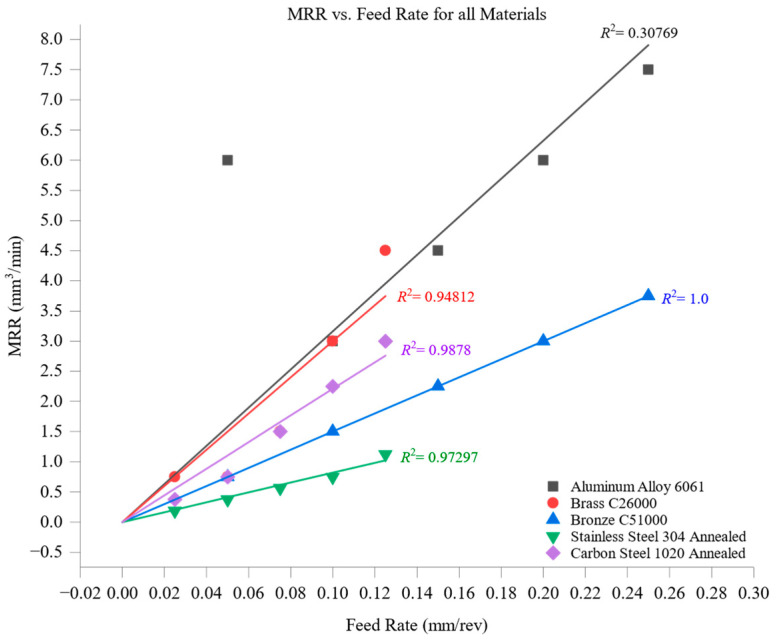
MRR vs. feed rate for all materials with their linear fitting.

Figure 1 illustrates the variation of material removal rate (MRR) with feed rate across different materials, highlighting the influence of thermal and mechanical properties on machining performance. The results demonstrate a consistent trend: as the feed rate increases, MRR rises for all materials due to the higher volume of material engaged in each cutting pass. However, the rate of increase and the absolute MRR values vary significantly based on material properties such as thermal conductivity, hardness, and machinability. Among the tested materials, aluminum alloy 6061 exhibited the highest MRR, peaking at approximately 7.5 mm^3^/min at the maximum feed rate of 0.25 mm/rev. This superior performance is attributed to aluminum’s exceptional thermal conductivity (205 W/m·K) and low hardness (95 HBW), which facilitate effective heat dissipation, reducing thermal stresses at the tool–workpiece interface. The soft nature of aluminum minimizes cutting resistance, allowing for efficient material shearing and removal, even at elevated feed rates. It is noted that the MRR at the lowest feed rate (0.05 mm/rev) for aluminum 6061 appears slightly elevated compared to the overall trend. This anomaly may be attributed to aluminum’s high thermal conductivity and low cutting resistance, which enable efficient material removal even at low cutting loads. Additionally, small variations in mass loss measurements have a proportionally larger effect at lower feed rates, contributing to the observed deviation, which remains within the measurement uncertainty range. Brass C26000 and bronze C51000 demonstrated intermediate MRR values, reaching peaks of approximately 4.5 mm^3^/min and 3.75 mm^3^/min, respectively. Despite having similar hardness levels (brass: 115 HBW, bronze: 112 HBW), the difference in their MRR behavior is primarily influenced by thermal conductivity—brass (109 W/m·K) dissipates heat more efficiently than bronze (60 W/m·K), reducing localized heating and enabling slightly better material removal performance. Both materials, however, exhibit moderate machinability, explaining their lower MRR compared to aluminum. Carbon steel 1020 exhibited a lower MRR, peaking at approximately 3.0 mm^3^/min at a feed rate of 0.125 mm/rev. Its higher hardness (150 HBW) and moderate thermal conductivity (51 W/m·K) contribute to increased cutting resistance and tool wear, thereby limiting the achievable MRR. Unlike brass and aluminum, carbon steel’s combination of mechanical strength and limited heat dissipation causes higher stress concentrations on the cutting tool, reducing overall efficiency. At the lower end of the spectrum, stainless steel 304 recorded the lowest MRR, peaking at approximately 1.125 mm^3^/min. The combination of low thermal conductivity (16 W/m·K) and high hardness (210 HBW) leads to significant thermal buildup in the cutting zone, promoting rapid tool wear and reducing the material removal efficiency. The heat accumulation in stainless steel results in work-hardening effects, further increasing cutting resistance and slowing down the removal process.

**Figure 2:**
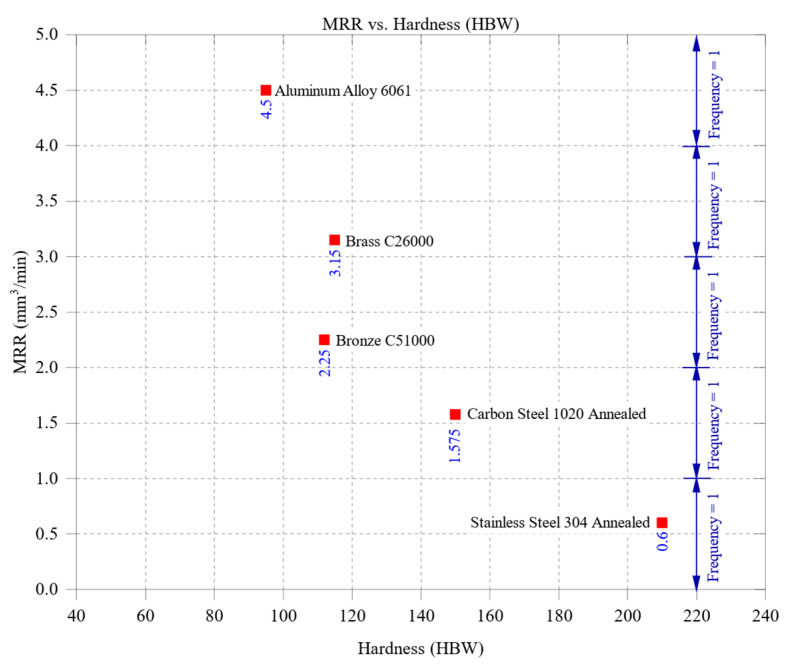
MRR vs. hardness (HBW).

Figure 2 illustrates the influence of material hardness (HBW) on the material removal rate (MRR), revealing a strong inverse relationship between these parameters. As material hardness increases, MRR generally declines due to higher cutting resistance, increased tool wear, and the reduced ability of the tool to penetrate the workpiece efficiently. Aluminum alloy 6061, the softest material among the tested samples, exhibited the highest MRR, reaching approximately 4.5 mm^3^/min. This superior material removal efficiency is primarily attributed to aluminum’s low cutting resistance and excellent machinability, allowing for rapid and efficient chip formation. Its high thermal conductivity (205 W/m·K) also ensures effective heat dissipation, preventing localized temperature buildup and reducing the likelihood of tool degradation, further enhancing machining performance. In contrast, brass C26000 and bronze C51000, with hardness values of 115 HBW and 112 HBW, respectively, demonstrated a moderate reduction in MRR, with brass reaching approximately 3.15 mm^3^/min and bronze about 2.25 mm^3^/min. Compared to aluminum, the slightly lower machinability of these materials results in higher cutting forces and more gradual material removal. Brass, with its relatively high thermal conductivity of 109 W/m·K, supports stable cutting conditions, but its increased hardness contributes to elevated tool wear, which reduces MRR. Bronze, with a slightly higher hardness and lower thermal conductivity (60 W/m·K), exhibits a further reduction in MRR due to its greater resistance to cutting and higher tendency for heat accumulation at the cutting zone. Carbon steel 1020, with a hardness of 150 HBW, presented a further decrease in MRR, reaching approximately 1.575 mm^3^/min. The combined effects of increased hardness and lower thermal conductivity (51 W/m·K) lead to greater cutting resistance, elevated temperatures at the tool–workpiece interface, and accelerated tool wear, all of which contribute to reduced material removal efficiency. The lowest MRR was observed for stainless steel 304, which has the highest hardness of 210 HBW and the lowest thermal conductivity of 16 W/m·K, yielding an MRR of approximately 0.6 mm^3^/min. The machining of stainless steel is significantly more challenging due to its poor heat dissipation, which results in excessive thermal stresses, rapid tool wear, and increased energy requirements for material removal. These characteristics make stainless steel a difficult material to machine efficiently without specialized tooling and optimized cutting parameters.

It is important to note that while this figure focuses on the influence of hardness, the observed trends are also affected by variations in thermal conductivity across materials. For example, aluminum 6061 has low hardness and the highest thermal conductivity among the tested materials, which enhances heat dissipation and further supports high MRR values. This interplay highlights the need to consider both thermal and mechanical properties when interpreting machining performance.

The overall findings from Figure 2 underscore the dominant role of material hardness in controlling MRR, where softer materials like aluminum exhibit high productivity due to their ease of cutting. In contrast, harder materials such as stainless steel require strategic machining approaches to maintain acceptable MRR levels. The results further highlight the importance of balancing thermal conductivity and hardness to optimize machining performance, as materials with better heat dissipation generally sustain more stable cutting conditions, reducing tool wear and improving overall efficiency.

**Figure 3:**
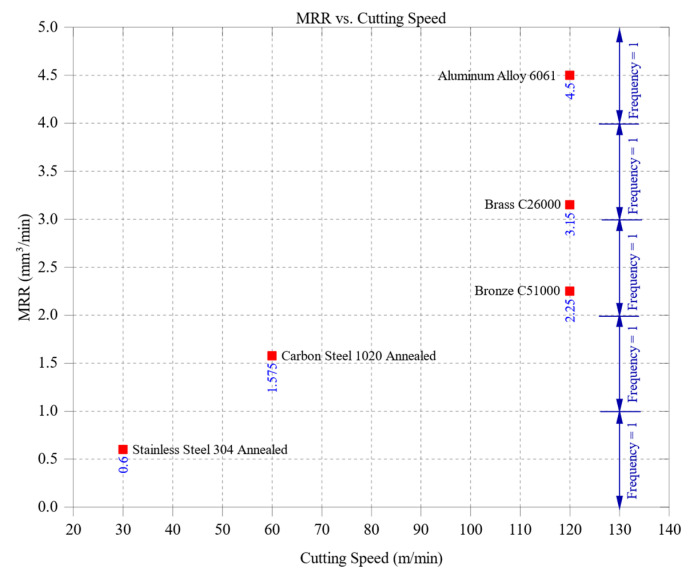
MRR vs. cutting speed.

Figure 3 illustrates the relationship between material removal rate (MRR) and cutting speed across the tested materials, demonstrating a general increase in MRR as cutting speed rises. This trend reflects the greater volume of material removed per unit time when the cutting tool moves faster, enhancing machining efficiency. Among the tested materials, aluminum alloy 6061 achieved the highest MRR values, peaking at approximately 4.5 mm^3^/min at the highest cutting speed of 120 m/min. This high removal rate is attributed to aluminum’s low hardness (95 HBW) and excellent machinability, which enable it to sustain efficient material removal even at elevated speeds. Its superior thermal conductivity (205 W/m·K) facilitates rapid heat dissipation, preventing excessive tool wear and ensuring stable cutting conditions, which further enhance its MRR performance. Brass C26000 and bronze C51000 displayed intermediate MRR values, with brass reaching approximately 3.15 mm^3^/min and bronze 2.25 mm^3^/min at 120 m/min. Their moderate hardness levels (brass: 115 HBW, bronze: 112 HBW) and balanced thermal properties (brass: 109 W/m·K, bronze: 60 W/m·K) contribute to steady material removal rates without excessive thermal stresses or rapid tool degradation. With its higher thermal conductivity, brass dissipates heat more effectively than bronze, leading to slightly higher MRR values, though both materials maintain relatively efficient machining performance. Carbon steel 1020, with a hardness of 150 HBW and thermal conductivity of 51 W/m·K, exhibited a maximum MRR of approximately 1.575 mm^3^/min at a 60 m/min cutting speed. The higher cutting resistance of carbon steel reduces the effectiveness of material removal at increased speeds, as greater forces are required to shear the material. Its lower thermal conductivity also contributes to heat accumulation at the cutting zone, accelerating tool wear and further restricting MRR. Stainless steel 304, the study’s hardest and least thermally conductive material, exhibited the lowest MRR values, peaking at approximately 0.6 mm^3^/min at 30 m/min. With a hardness of 210 HBW and thermal conductivity of just 16 W/m·K, stainless steel generates significant cutting resistance and localized heating, increasing tool wear and limiting material removal efficiency. These factors make machining stainless steel more challenging, requiring conservative cutting speeds to balance MRR with tool longevity.

Overall, Figure 3 highlights the significant impact of material properties on the effectiveness of increasing cutting speed. Softer, more thermally conductive materials like aluminum achieve greater productivity at higher speeds due to their lower cutting resistance and superior heat dissipation. Although the trends in this figure emphasize the effect of increasing cutting speed, the results are also modulated by differences in material thermal conductivity and hardness. Materials with higher thermal conductivity (e.g., aluminum 6061 and brass C26000) are better able to maintain machining stability at higher speeds, whereas harder, low-conductivity materials exhibit thermal stress and reduced MRR. Thus, material properties should be considered alongside speed when interpreting these results. In contrast, harder materials like stainless steel and carbon steel require more controlled machining conditions to prevent excessive tool wear and maintain machining stability. These findings emphasize the necessity of selecting appropriate cutting speeds based on material characteristics to optimize machining efficiency while ensuring tool durability and process stability.

**Figure 4:**
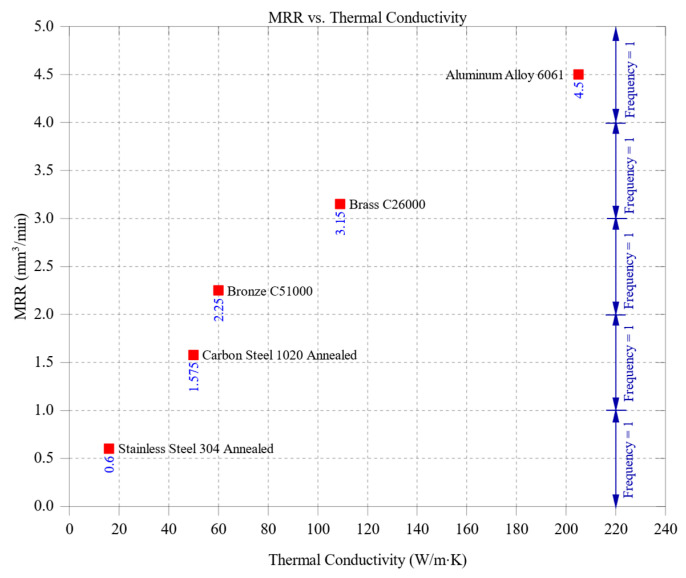
MRR vs. thermal conductivity.

Figure 4 illustrates the relationship between thermal conductivity and material removal rate (MRR), highlighting a clear correlation where materials with higher thermal conductivity generally achieve higher MRR values due to improved heat dissipation and reduced thermal stress during machining. Aluminum alloy 6061, possessing the highest thermal conductivity of 205 W/m·K, recorded the highest MRR, peaking at approximately 4.5 mm^3^/min. This superior performance is attributed to aluminum’s ability to dissipate heat efficiently, which prevents excessive thermal buildup at the cutting zone. By maintaining stable thermal conditions, aluminum minimizes tool wear, enhances cutting stability, and allows for sustained high-speed machining without compromising precision or surface integrity. Brass C26000, with a thermal conductivity of 109 W/m·K, and bronze C51000, at 60 W/m·K, demonstrated intermediate MRR values of approximately 3.15 mm^3^/min and 2.25 mm^3^/min, respectively. These materials benefit from moderate thermal conductivity, allowing for stable machining conditions while preventing excessive heat accumulation. However, their heat dissipation capabilities are less effective than aluminum, which limits their maximum MRR compared to softer, more thermally conductive materials. Despite brass’s relatively high thermal conductivity, its hardness of 115 HBW introduces additional cutting resistance, explaining its slightly lower MRR than aluminum. Bronze, having similar hardness (112 HBW) and being less thermally conductive than brass, exhibited a further reduction in MRR, reflecting its higher energy requirements for efficient cutting. Carbon steel 1020, with a thermal conductivity of 51 W/m·K, achieved a lower MRR of about 1.575 mm^3^/min. The material’s limited ability to dissipate heat results in increased temperature buildup in the cutting zone, accelerating tool wear and thermal deformation. This, in turn, reduces machining efficiency and requires more conservative feed rates and cutting speeds to maintain process stability. Stainless steel 304, with the lowest thermal conductivity of 16 W/m·K, exhibited the lowest MRR, peaking at approximately 0.6 mm^3^/min. Stainless steel’s inability to dissipate heat efficiently leads to significant thermal stress and cutting resistance, contributing to rapid tool wear and reduced material removal efficiency. This characteristic makes machining stainless steel more challenging, requiring lower cutting speeds and optimized cooling strategies to mitigate thermal damage.

Overall, Figure 4 highlights the substantial influence of thermal conductivity on machining efficiency. Materials with higher thermal conductivity, such as aluminum, facilitate faster material removal by maintaining stable cutting conditions, whereas lower-thermal-conductivity materials, such as stainless steel, impose greater machining constraints due to excessive heat accumulation. These findings reinforce the need to optimize machining parameters based on a material’s thermal properties to maximize productivity while minimizing tool wear and thermal stress.

While Figure 4 demonstrates a clear positive correlation between thermal conductivity and MRR, it is important to recognize that material hardness simultaneously influences the outcome. For instance, although brass C26000 and bronze C51000 have similar hardness, their differing thermal conductivities lead to distinct MRR behaviors. In contrast, stainless steel 304 has low thermal conductivity and hardness, compounding its poor machinability. These combined effects illustrate the limitations of interpreting thermal conductivity in isolation and reinforce the importance of the multivariate regression model presented later in Section 4.3.

### 4.2. Influence of Machining Parameters on Surface Integrity

**Figure 5:**
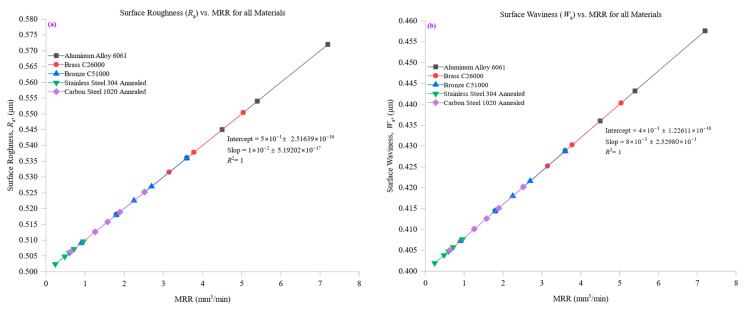
(**a**) Surface roughness (*R*_a_) and (**b**) surface waviness (*W*_a_) vs. MRR for all materials.

Figure 5a investigates the relationship between surface roughness (*R*_a_) and material removal rate (MRR) across the tested materials, revealing a trend where surface roughness tends to increase with MRR, although the rate of increase varies depending on material properties. Aluminum alloy 6061, known for its high thermal conductivity (205 W/m·K) and low hardness (95 HBW), demonstrated the lowest *R*_a_ values across all MRR levels. At an MRR of 1.8 mm^3^/min, *R*_a_ measured approximately 0.518 µm, and even at the highest MRR of 7.2 mm^3^/min, *R*_a_ only increased slightly to 0.572 µm. The minimal variation in *R*_a_ with increasing MRR indicates that aluminum maintains excellent surface integrity under varying cutting conditions. This behavior can be attributed to aluminum’s efficient heat dissipation, stabilizing cutting forces, and minimizing tool wear, which ensures consistent material removal with minimal surface degradation. Brass C26000 and bronze C51000, with intermediate thermal conductivities (109 W/m·K and 60 W/m·K, respectively) and moderate hardness values (115 HBW and 112 HBW), exhibited slightly higher *R*_a_ values than aluminum but maintained stable surface conditions across different MRR levels. For brass, *R*_a_ increased from 0.5126 µm at 1.26 mm^3^/min to 0.5504 µm at 5.04 mm^3^/min, while for bronze, *R*_a_ rose from 0.509 µm at 0.9 mm^3^/min to 0.536 µm at 3.6 mm^3^/min. The controlled increase in roughness with higher MRR reflects their balanced machinability, as moderate thermal conductivity aids in heat dissipation while moderate hardness prevents excessive surface damage. Carbon steel 1020, with a thermal conductivity of 51 W/m·K and a hardness of 150 HBW, demonstrated an increase in *R*_a_ from 0.5063 µm at an MRR of 0.63 mm^3^/min to 0.5252 µm at 2.52 mm^3^/min. The rise in roughness is more pronounced compared to aluminum, brass, and bronze, reflecting the influence of carbon steel’s higher cutting resistance and lower heat dissipation capacity. These factors lead to greater cutting forces and localized thermal buildup, promoting tool wear and surface irregularities and increasing *R*_a_ at higher MRR values. Stainless steel 304, the hardest material tested (210 HBW) and the least thermally conductive (16 W/m·K), exhibited the highest *R*_a_ values across all MRR levels. At an MRR of 0.24 mm^3^/min, *R*_a_ measured 0.5024 µm, increasing to 0.5096 µm at 0.96 mm^3^/min. The relatively high increase in roughness with MRR highlights the challenges associated with machining stainless steel, where poor heat dissipation leads to excessive thermal stress and tool wear. These factors contribute to rougher surfaces as machining conditions become more aggressive.

Figure 5b extends this analysis to surface waviness (*W*_a_) and its relationship with MRR, revealing how large-scale surface undulations evolve with different machining conditions. Aluminum alloy 6061, with its high thermal conductivity and low hardness, exhibited the lowest *W*_a_ values, maintaining excellent waviness control. At an MRR of 1.8 mm^3^/min, *W*_a_ was approximately 0.4144 µm, increasing only slightly to 0.4576 µm at the highest MRR of 7.2 mm^3^/min. The small increase in *W*_a_ demonstrates aluminum’s ability to maintain a smooth waviness profile even at high material removal rates, attributed to its efficient heat dissipation and low cutting resistance. Brass C26000 and bronze C51000 exhibited moderate increases in *W*_a_ with rising MRR. Brass showed an increase from 0.41008 µm at 1.26 mm^3^/min to 0.44032 µm at 5.04 mm^3^/min, while bronze increased from 0.4072 µm at 0.9 mm^3^/min to 0.4288 µm at 3.6 mm^3^/min. These materials’ intermediate thermal conductivity helps mitigate excessive thermal buildup, resulting in relatively stable waviness profiles. However, their slightly higher hardness than aluminum leads to greater cutting resistance, contributing to a more noticeable increase in *W*_a_ at higher MRR values. Carbon steel 1020, with its lower thermal conductivity and higher hardness, exhibited more significant increases in *W*_a_ as MRR rose. *W*_a_ values increased from 0.40504 µm at an MRR of 0.63 mm^3^/min to 0.42016 µm at 2.52 mm^3^/min. The increased waviness can be attributed to carbon steel’s higher cutting forces and reduced ability to dissipate heat, leading to greater surface deformation at higher material removal rates. Stainless steel 304, the hardest and least thermally conductive material, exhibited the highest *W*_a_ values and the sharpest increase in waviness. At an MRR of 0.24 mm^3^/min, *W*_a_ measured 0.40192 µm, rising to 0.40768 µm at 0.96 mm^3^/min. The rapid increase in *W*_a_ with higher MRR is due to stainless steel’s poor thermal conductivity and high cutting resistance, which lead to pronounced cutting vibrations, tool wear, and uneven material removal. The resultant surface undulations are more significant, reflecting stainless steel’s tendency to develop higher waviness under aggressive machining conditions.

In summary, Figure 5 emphasizes material properties’ fundamental influence on surface roughness and waviness during machining. Materials with high thermal conductivity and low hardness, such as aluminum alloy 6061, maintain stable surface quality with minimal increases in *R*_a_ and *W*_a_ across all MRR levels. In contrast, harder and less thermally conductive materials, such as stainless steel 304, experience substantial increases in both roughness and waviness due to greater thermal stress, cutting resistance, and tool wear. These findings underscore the necessity of optimizing machining parameters for different materials to achieve the best balance between productivity and surface quality.

**Figure 6:**
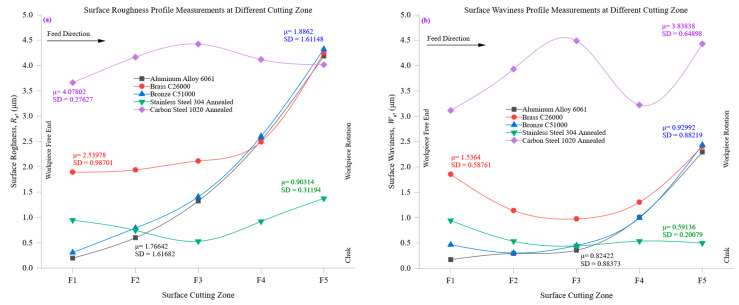
(**a**) Surface roughness and (**b**) surface waviness at different cutting zones.

Figure 6a provides a detailed evaluation of surface roughness (*R*_a_) at different cutting zones across the tested materials, shedding light on how material properties influence roughness progression. Aluminum alloy 6061, characterized by its high thermal conductivity (205 W/m·K) and low hardness (95 HBW), exhibited the lowest *R*_a_ values across all cutting zones, maintaining a consistent roughness profile. The *R*_a_ values ranged between 0.52 µm at the initial stage and 0.58 µm at later stages, with only a minimal increase over successive cutting zones. This stability is attributed to aluminum’s excellent heat dissipation, which prevents localized thermal stresses and ensures steady cutting performance. Its machinability allows for smoother material removal, producing a refined and consistent surface texture. Stainless steel 304, in contrast, demonstrated the highest surface roughness values across all cutting zones due to its low thermal conductivity (16 W/m·K) and high hardness (210 HBW). The initial *R*_a_ values were around 0.92 µm but increased progressively, surpassing 1.0 µm at deeper cutting zones. High cutting resistance, rapid tool wear, and poor heat dissipation lead to more pronounced surface irregularities. The accumulation of thermal energy in the cutting region causes material hardening effects, further deteriorating surface quality and amplifying roughness variations between cutting zones. Intermediate materials, such as brass C26000 and bronze C51000, exhibited moderately roughness profiles. Brass, with a thermal conductivity of 109 W/m·K and hardness of 115 HBW, started with an *R*_a_ of approximately 0.65 µm, which increased slightly as the cutting depth progressed. Bronze displayed a similar trend, beginning with an *R*_a_ of about 0.71 µm and increasing modestly across cutting zones. These materials balance thermal dissipation and mechanical hardness, preventing excessive roughness while still demonstrating slight variations in texture. Carbon steel 1020, with a thermal conductivity of 51 W/m·K and a hardness of 150 HBW, displayed roughness characteristics between those of the softer alloys and harder stainless steel. The *R*_a_ values were initially around 0.76 µm and increased slightly to approximately 0.8 µm at deeper cutting zones. While carbon steel exhibits higher cutting resistance than brass or bronze, its moderate machinability ensures relatively stable surface quality compared to stainless steel. Overall, Figure 6a highlights that materials with high thermal conductivity and low hardness, such as aluminum, maintain smoother surfaces across all cutting zones due to efficient heat management and stable cutting conditions. Conversely, harder and less thermally conductive materials, like stainless steel, experience greater surface deterioration as cutting progresses, necessitating careful control of machining parameters to mitigate surface degradation.

Figure 6b investigates the evolution of surface waviness (*W*_a_) across different cutting zones, providing insights into the macroscopic surface undulations resulting from machining. The results reveal that materials with higher thermal conductivity and lower hardness exhibit significantly lower waviness levels, while harder and less thermally conductive materials develop more pronounced undulations as cutting progresses. Aluminum alloy 6061 exhibited the lowest *W*_a_ values across all cutting zones, with an initial *W*_a_ of approximately 0.4144 µm, which increased marginally to 0.4576 µm in later zones. The material’s high thermal conductivity facilitates efficient heat dissipation, reducing the likelihood of thermal distortions and ensuring stable cutting conditions. This results in minimal waviness variation, reflecting aluminum’s excellent machinability and ability to sustain consistent surface quality. Although stainless steel 304 did not exhibit the highest absolute *W*_a_ values, it showed a relatively sharper response to cutting progression, with *W*_a_ values increasing from approximately 0.4019 µm to 0.4077 µm. The material’s high hardness (210 HBW) and low thermal conductivity (16 W/m·K) lead to greater cutting forces and localized thermal stresses, promoting surface distortions and waviness amplification. The accumulation of heat near the cutting region results in increased tool wear and cutting instability, exacerbating the formation of macroscopic undulations. Intermediate materials, such as brass C26000 and bronze C51000, displayed moderate waviness characteristics. Brass exhibited an initial *W*_a_ of around 1.06 µm, which increased slightly as the cutting process advanced. Bronze, with a slightly lower thermal conductivity than brass, followed a similar trend, with *W*_a_ increasing from 1.12 µm to approximately 1.18 µm. Due to their mechanical properties and machining response, these materials maintain more stable waviness profiles than stainless steel but are not as smooth as aluminum. Carbon steel 1020 exhibited a moderate waviness progression, with *W*_a_ values beginning at approximately 1.27 µm and increasing to around 1.36 µm. The material’s combination of intermediate hardness and thermal conductivity resulted in a more stable surface profile than stainless steel but one less uniform than aluminum. Heat accumulation during machining led to minor undulations, reflecting the material’s higher cutting resistance than softer alloys.

In summary, Figure 6b emphasizes the significant influence of material properties on surface waviness at different cutting zones. Softer, highly conductive materials such as aluminum exhibit minimal waviness variations, while harder and less thermally conductive materials like stainless steel experience increasing waviness as machining progresses. These findings reinforce the importance of optimizing cutting conditions and tool selection to minimize surface waviness, particularly for harder materials with low thermal conductivity.

**Figure 7:**
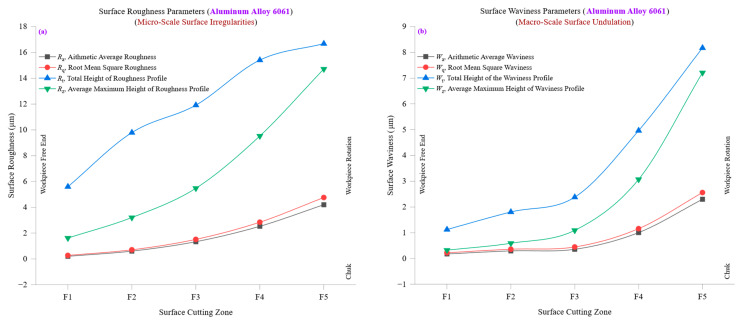
(**a**) Surface roughness parameters and (**b**) surface waviness parameters for aluminum alloy 6061.

Figure 7a presents the evolution of surface roughness parameters—average roughness (*R*_a_), root mean square roughness (*R*_q_), total height of the roughness profile (*R*_t_), and ten-point mean roughness (*R*_z_)—across different cutting zones in aluminum alloy 6061. The progressive variation of these parameters provides insights into how surface texture develops during machining, reflecting the influence of tool wear, cutting stability, and thermal effects. At the initial cutting zone (F1), *R*_a_ and *R*_q_ values are relatively low, with *R*_a_ at approximately 0.1971 µm and *R*_q_ at 0.2633 µm, indicating a highly polished and uniform surface. These values suggest that the tool is in optimal condition at the early machining stages, allowing for minimal roughness formation. However, *R*_a_ increases significantly as machining progresses, reaching 4.189 µm at F5, while *R*_q_ peaks at 4.7497 µm. This rise in roughness is primarily due to progressive tool wear and increased cutting resistance, which generates microstructural deformations and surface irregularities. The total height of the roughness profile (*R*_t_) and ten-point mean roughness (*R*_z_) further illustrate the deterioration of surface texture over multiple cutting zones. At F1, *R*_t_ is approximately 1.6079 µm, while *R*_z_ is also 1.6079 µm, suggesting minimal peak-to-valley height variations in the machined surface. However, by the final cutting zone (F5), *R*_t_ reaches 14.7138 µm, with *R*_z_ following a similar trend, reflecting the development of deeper surface grooves and irregularities. The increase in *R*_t_ and *R*_z_ is attributed to the accumulation of machining-induced strain and tool degradation, leading to pronounced peaks and valleys on the surface. The trends in *R*_a_, *R*_q_, *R*_t_, and *R*_z_ collectively indicate that while roughness increases over time due to progressive tool wear, the uniformity of the roughness profile remains relatively stable. The variations observed suggest that while aluminum alloy 6061 maintains good machinability, extended cutting operations require optimized machining parameters to minimize roughness buildup and maintain high surface integrity.

Figure 7b investigates the variation of surface waviness parameters, average waviness (*W*_a_), root mean square waviness (*W*_q_), total height of the waviness profile (*W*_t_), and ten-point mean waviness (*W*_z_) across different cutting zones, offering insights into large-scale surface undulations that develop during machining. At the initial cutting zone (F1), aluminum alloy 6061 exhibits low waviness values, with *W*_a_ at approximately 0.1733 µm and *W*_q_ at 0.222 µm. These values indicate a highly uniform and smooth waviness profile, with minimal deviations caused by cutting instabilities. As machining progresses, *W*_a_ increases significantly to 2.2958 µm at F5, while *W*_q_ follows a similar trend, reaching 2.5575 µm. This increase is primarily attributed to cumulative tool wear, slight variations in tool engagement, and material deformation effects, which introduce larger-scale undulations across the surface. The total height of the waviness profile (*W*_t_) and ten-point mean waviness (*W*_z_) provide further insight into the evolving waviness characteristics. At F1, *W*_t_ is 1.1216 µm, while *W*_z_ is 0.3227 µm, indicating a stable and consistent waviness pattern with minimal variations. However, by the final cutting zone (F5), *W*_t_ increases to 8.1708 µm, while *W*_z_ reaches 7.2095 µm, confirming the development of significant surface undulations. These pronounced variations result from thermal expansion, tool deflection, and increased vibrations, which contribute to irregular waviness patterns, particularly in extended machining cycles. The increasing trends in *W*_a_, *W*_q_, *W*_t_, and *W*_z_ reinforce the role of thermal conductivity and cutting stability in controlling surface waviness. Aluminum’s high thermal conductivity (205 W/m·K) helps dissipate heat efficiently, reducing thermal-stress-induced waviness. However, at higher cutting zones, machining vibrations and accumulated tool wear begin to dominate, leading to noticeable waviness variations.

The combined trends observed in Figure 7a,b suggest a strong interdependence between roughness and waviness evolution during machining. While surface roughness parameters (*R*_a_, *R*_q_, *R*_t_, *R*_z_) primarily reflect fine-scale irregularities influenced by direct tool–material interactions, surface waviness parameters (*W*_a_, *W*_q_, *W*_t_, *W*_z_) capture larger-scale deviations that emerge due to cutting vibrations, tool wear progression, and thermal expansion. At lower cutting zones, both roughness and waviness parameters remain low, indicating stable machining conditions with minimal deviations. However, as cutting progresses, roughness and waviness values increase significantly, with roughness showing a gradual increase, whereas waviness exhibits larger, more abrupt changes at higher cutting zones. This divergence suggests that roughness increases steadily as tool wear accumulates, whereas waviness is more sensitive to external machining instabilities, such as vibration and heat accumulation. The increasing trends in *R*_a_ and *W*_a_ suggest that as tool wear progresses, both micro-scale roughness and macro-scale waviness intensify, emphasizing the need for tool condition monitoring and adaptive cutting strategies. The steady rise in *R*_t_ and *W*_t_ highlights peak-to-valley deviations’ growing influence on fine and coarse surface features, reinforcing the importance of maintaining optimal cutting forces to limit excessive deformation.

Figure 7a,b demonstrate how surface roughness and waviness evolve across different cutting zones in aluminum alloy 6061. While *R*_a_, *R*_q_, *R*_t_, and *R*_z_ reflect fine-scale roughness progression due to tool wear and cutting forces, *W*_a_, *W*_q_, *W*_t_, and *W*_z_ capture large-scale waviness variations caused by thermal and mechanical instabilities. The findings underscore the necessity of optimizing machining parameters to balance roughness and waviness control, ensuring superior surface quality in CNC-turned aluminum components. The correlation between roughness and waviness trends suggests that while both parameters increase over time, waviness is more susceptible to external factors such as vibrations and thermal expansion, necessitating real-time machining adjustments to maintain high-quality surface finishes.

**Figure 8:**
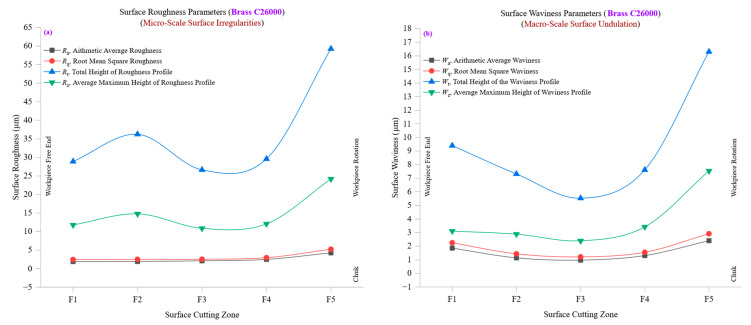
(**a**) Surface roughness parameters and (**b**) surface waviness parameters for brass C26000.

Figure 8a presents the variation in surface roughness parameters, average roughness (*R*_a_), root mean square roughness (*R*_q_), total height of the roughness profile (*R*_t_), and ten-point mean roughness (*R*_z_) across different cutting zones in brass C26000, revealing critical trends in surface texture development during machining. At the initial cutting zone (F1), *R*_a_ starts at approximately 1.8958 µm, with *R*_q_ at 2.4427 µm, indicating a relatively smooth surface with minimal roughness variations. At this stage, the Rq/Ra ratio of 1.288 suggests a consistent surface texture with a balanced distribution of peaks and valleys. However, as machining progresses, *R*_a_ increases steadily, reaching 4.2544 µm at F5, while *R*_q_ peaks at 5.2215 µm, indicating an accumulation of microstructural deformations and irregularities due to prolonged tool–material interactions. The total height of the roughness profile (*R*_t_) and ten-point mean roughness (*R*_z_) further demonstrate the impact of machining on surface integrity. At F1, *R*_t_ is approximately 11.78 µm, while *R*_z_ follows closely at 11.78 µm, indicating minimal peak-to-valley height deviations in the machined surface. However, by the final cutting zone (F5), *R*_t_ reaches 24.18 µm, with *R*_z_ mirroring this increase, reflecting deeper grooves and more prominent surface irregularities. This sharp rise in *R*_t_ and *R*_z_ suggests that increased tool wear, higher cutting forces, and localized material deformations contribute to surface degradation as the cutting process continues. The *R*_q_/*R*_a_ ratio remains reasonably stable, with values fluctuating between 1.18 and 1.29 across cutting zones, reinforcing the notion that while roughness increases, the overall texture remains relatively uniform. However, the gradual rise in *R*_sk_ (skewness) from −0.55 at F1 to 0.41 at F5 indicates that as machining progresses, the surface transitions from one dominated by valleys to one where sharp peaks become more prevalent. This change is a direct consequence of tool wear, increased strain hardening, and higher cutting resistance, which result in pronounced peaks and reduced material ductility.

Figure 8b explores the evolution of waviness parameters, average waviness (*W*_a_), root mean square waviness (*W*_q_), total waviness height (*W*_t_), and ten-point mean waviness (*W*_z_), across different cutting zones, providing a broader perspective on larger-scale surface deviations during machining. At the initial cutting zone (F1), *W*_a_ is approximately 1.86 µm, with *W*_q_ at 2.24 µm, reflecting a relatively stable and uniform waviness profile. The *W*_q_/*W*_a_ ratio of 1.21 suggests a moderately consistent waviness pattern with minimal fluctuations in undulations across the surface. However, as machining progresses, *W*_a_ increases gradually to 2.40 µm at F5. In comparison, *W*_q_ peaks at 2.91 µm indicate that larger-scale surface undulations emerge due to accumulated tool deflections, thermal expansion, and cutting vibrations. The total waviness height (*W*_t_) and ten-point mean waviness (*W*_z_) provide additional insights into the macro-scale variations in surface texture. At the early stages (F1), *W*_t_ is 9.39 µm, while *W*_z_ is 3.10 µm, signifying controlled and stable waviness characteristics. However, by the final cutting zone (F5), *W*_t_ rises significantly to 16.30 µm, while *W*_z_ increases to 7.54 µm, highlighting the progressive formation of pronounced waviness features. These changes can be attributed to thermal expansion effects, variations in tool-chip interaction, and increasing cutting resistance, all of which contribute to greater fluctuations in the overall surface profile. An interesting trend is observed in waviness skewness (*W*_sk_) and waviness kurtosis (*W*_ku_). Initially, *W*_sk_ is −0.34 at F1, indicating a surface dominated by valleys, but as machining progresses, *W*_sk_ moves toward −0.20 at F5, suggesting a shift toward a more balanced surface with a mix of peaks and valleys. The *W*_ku_ values, which start at 2.34 at F1 and increase to 2.93 at F5, indicate a gradual sharpening of the waviness distribution, reinforcing the idea that machining dynamics increasingly influence surface undulations as cutting progresses.

A direct correlation exists between surface roughness (*R*_a_, *R*_q_, *R*_t_, *R*_z_) and waviness (*W*_a_, *W*_q_, *W*_t_, *W*_z_), demonstrating how micro-scale roughness progression is linked to macro-scale waviness fluctuations during machining. At lower cutting zones (F1–F2), both roughness and waviness parameters remain relatively low, suggesting stable cutting conditions, minimal tool wear, and effective heat dissipation. However, roughness and waviness values increase significantly at higher cutting zones (F4–F5), reflecting the combined influence of tool wear, material deformation, and machining-induced vibrations. The trends observed in *R*_q_/*R*_a_ and *W*_q_/*W*_a_ ratios confirm that while roughness develops progressively, waviness is more susceptible to sudden variations caused by external machining factors. The increasing *R*_t_ and *W*_t_ values further emphasize that as machining progresses, peak-to-valley variations in both fine and coarse surface features become more pronounced, reinforcing the importance of maintaining optimal cutting conditions to prevent excessive surface defects.

Figure 8a,b highlight the evolution of surface roughness and waviness across different cutting zones for brass C26000, demonstrating how machining conditions affect both micro-scale and macro-scale surface characteristics. While *R*_a_, *R*_q_, *R*_t_, and *R*_z_ provide insights into the fine-scale texture influenced by tool–material interactions and strain hardening, *W*_a_, *W*_q_, *W*_t_, and *W*_z_ capture larger-scale surface undulations driven by thermal expansion, tool deflections, and machining dynamics. These findings underscore the importance of machining parameter optimization to balance roughness and waviness control, ensuring high surface integrity in CNC-turned brass components. The results further suggest that while brass exhibits stable cutting behavior, prolonged machining cycles necessitate close monitoring of tool wear and thermal effects to maintain superior surface quality.

**Figure 9:**
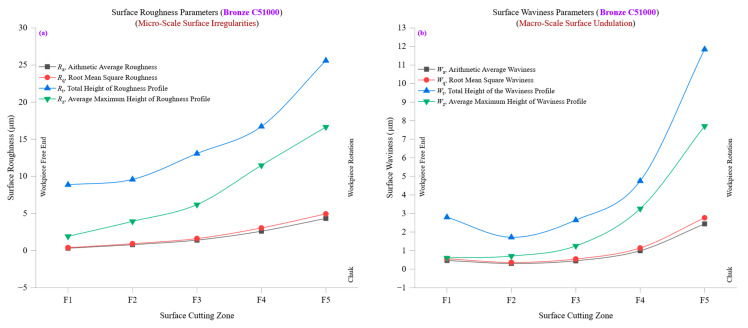
(**a**) Surface roughness parameters and (**b**) surface waviness parameters for bronze C51000.

Figure 9a presents a detailed examination of surface roughness parameters, including average roughness (*R*_a_), root mean square roughness (*R*_q_), total height of the roughness profile (*R*_t_), and ten-point mean roughness (*R*_z_) across various cutting zones in bronze C51000. The findings reveal a progressive increase in roughness parameters as machining progresses, indicative of tool wear accumulation, strain hardening, and thermal effects that influence the material’s response to cutting. At the initial cutting zone (F1), *R*_a_ is approximately 0.3066 µm, while *R*_q_ is 0.3833 µm, signifying a relatively smooth surface with minimal variations. The *R*_q_/*R*_a_ ratio at this stage is 1.25, indicating a well-balanced roughness distribution with minimal irregularities. However, as machining advances, *R*_a_ increases steadily to 4.3256 µm at F5, while *R*_q_ reaches 4.9391 µm, reflecting an increasingly coarse surface with a greater presence of microscopic deformations. The progressive rise in *R*_a_ and *R*_q_ directly results from increased tool wear, higher cutting forces, and gradual deterioration of the cutting edge, which contribute to greater surface irregularities. The total height of the roughness profile (*R*_t_) and ten-point mean roughness (*R*_z_) further illustrate the impact of machining on surface texture. At F1, *R*_t_ is around 1.91 µm, and *R*_z_ follows a similar pattern, indicating a highly uniform and consistent surface. However, by the final cutting zone (F5), *R*_t_ increases significantly to 16.65 µm, while *R*_z_ shows a similar peak, reflecting the formation of deeper peaks and valleys. This substantial increase in *R*_t_ and *R*_z_ suggests that as the cutting process continues, increased mechanical stress, material hardening, and localized deformation cause more pronounced roughness peaks, necessitating improved tool condition monitoring to maintain surface integrity. A closer look at *R*_q_/*R*_a_ ratios reveals that while roughness increases, the relative balance between *R*_q_ and *R*_a_ remains stable, fluctuating between 1.14 and 1.25. This consistency suggests that while machining-induced roughness increases progressively, the overall texture remains relatively homogeneous, with no abrupt changes in peak-to-valley distribution. However, an important observation is the steady rise in skewness (*R*_sk_) values, from −0.59 at F1 to 0.47 at F5, indicating a transition from valley-dominated surfaces to peak-dominated ones. This shift indicates increasing material deformation, tool-wear-induced plowing, and changes in chip formation dynamics, all of which play a role in shaping surface roughness over time.

Figure 9b provides an in-depth analysis of waviness parameters, including average waviness (*W*_a_), root mean square waviness (*W*_q_), total waviness height (*W*_t_), and ten-point mean waviness (*W*_z_) across different cutting zones in bronze C51000. The results demonstrate how macro-scale surface undulations evolve as machining progresses, influenced by factors such as thermal expansion, tool deflections, and cutting vibrations. At the initial cutting zone (F1), *W*_a_ is approximately 0.4653 µm, with *W*_q_ at 0.5605 µm, indicating minimal waviness variations. The *W*_q_/*W*_a_ ratio of 1.20 suggests a well-balanced waviness distribution with relatively smooth transitions between peaks and valleys. However, as machining continues, *W*_a_ increases steadily to 2.4367 µm at F5, while *W*_q_ reaches 2.7657 µm, reflecting a greater presence of larger-scale surface undulations. This increase in waviness is a direct result of thermal expansion effects, tool wear progression, and increasing dynamic instabilities during machining. The total waviness height (*W*_t_) and ten-point mean waviness (*W*_z_) provide additional insights into the progression of surface undulations. At F1, *W*_t_ is around 2.80 µm, and *W*_z_ is 0.60 µm, indicating a relatively uniform waviness profile. However, by the final cutting zone (F5), *W*_t_ rises dramatically to 11.84 µm, while *W*_z_ increases to 7.70 µm, signifying the development of more prominent waviness peaks and valleys. These changes reflect the combined influence of heat accumulation, cutting resistance, and increased material displacement due to prolonged machining exposure. A crucial observation is the behavior of waviness skewness (*W*_sk_) and waviness kurtosis (*W*_ku_) over different cutting zones. Initially, *W*_sk_ is −0.37 at F1, indicating a valleys-dominated surface. However, as machining progresses, *W*_sk_ stabilizes near zero, suggesting a transition toward a more symmetric waviness distribution, where both peaks and valleys contribute equally to the overall surface profile. This transition can be attributed to machining-induced plastic deformation, which gradually modifies the surface profile and alters peak–valley distributions. The *W*_ku_ values, which start at 2.68 at F1 and increase slightly to 1.86 at F5, indicate that waviness distributions become less sharp over time as thermal and mechanical effects smooth out extreme undulations.

The correlation between surface roughness (*R*_a_, *R*_q_, *R*_t_, *R*_z_) and waviness (*W*_a_, *W*_q_, *W*_t_, *W*_z_) provides valuable insights into how micro-scale roughness development influences macro-scale waviness formation. At lower cutting zones (F1–F2), roughness and waviness values remain relatively low, indicating stable machining conditions with minimal tool wear or thermal distortion. However, at higher cutting zones (F4–F5), both roughness and waviness increase sharply, suggesting that the accumulation of cutting forces, tool wear, and heat generation leads to greater surface irregularities on both the micro and macro scales. The increasing *R*_q_/*R*_a_ and *W*_q_/*W*_a_ ratios confirm that while roughness progressively increases, waviness undergoes more dramatic shifts due to external machining factors. This reinforces the idea that while tool–material interactions and chip formation mechanics govern fine-scale surface roughness variations, larger-scale waviness is more susceptible to dynamic machining instabilities such as tool vibrations, heat-induced distortions, and machine rigidity effects.

The significant rise in *R*_t_ and *W*_t_ values at later cutting zones further emphasizes that as machining progresses, the combination of tool wear and thermal effects results in more pronounced peak-to-valley variations in both fine and coarse surface features. This highlights the importance of optimizing cutting conditions, selecting appropriate tool materials, and implementing real-time process monitoring to mitigate excessive roughness and waviness formation.

Figure 9a,b comprehensively analyze surface roughness and waviness evolution in bronze C51000, demonstrating how machining conditions influence both micro-scale and macro-scale surface characteristics. The findings reveal that while *R*_a_, *R*_q_, *R*_t_, and *R*_z_ provide critical insights into fine-scale texture development, *W*_a_, *W*_q_, *W*_t_, and *W*_z_ offer a broader perspective on large-scale undulations and waviness progression. These results emphasize the importance of balancing machining parameters to control both roughness and waviness, ensuring high surface integrity in CNC-turned bronze components. While bronze exhibits moderate machining stability, prolonged cutting cycles significantly increase roughness and waviness, requiring close monitoring of tool condition, cutting temperature, and material response.

**Figure 10:**
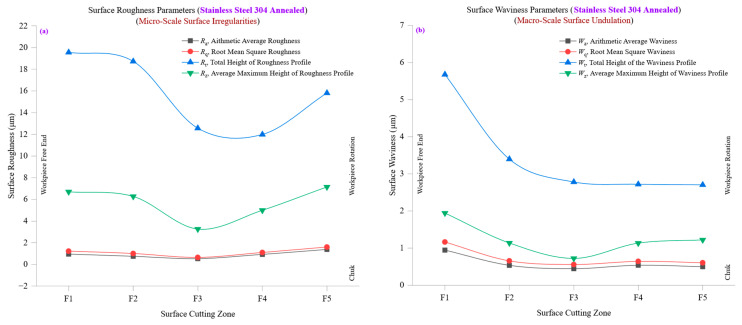
(**a**) Surface roughness parameters and (**b**) surface waviness parameters for stainless steel 304 annealed.

Figure 10a presents the progression of surface roughness parameters, including average roughness (*R*_a_), root mean square roughness (*R*_q_), total height of the roughness profile (*R*_t_), and ten-point mean roughness (*R*_z_) across multiple cutting zones in stainless steel 304 annealed. The data reveal a steady increase in roughness parameters with machining progression, demonstrating the material’s susceptibility to work hardening, tool wear effects, and thermal stress accumulation as cutting advances. At the initial cutting zone (F1), *R*_a_ is approximately 0.9465 µm, while *R*_q_ is 1.2199 µm, reflecting a moderately rough surface with minor irregularities. The *R*_q_/*R*_a_ ratio at this stage is 1.29, indicating a well-distributed roughness pattern with a moderate mix of peaks and valleys. However, as machining progresses, *R*_a_ increases to 1.3747 µm at F5. In comparison, *R*_q_ reaches 1.5979 µm, highlighting the gradual degradation of surface quality due to increasing tool wear, rising cutting forces, and thermal stress buildup. The total height of the roughness profile (*R*_t_) and ten-point mean roughness (*R*_z_) further illustrate how machining conditions affect surface texture evolution. Initially, *R*_t_ is around 6.69 µm, and *R*_z_ follows a similar trend, indicating a reasonably stable cutting environment. However, at the final cutting zone (F5), *R*_t_ increases to 7.14 µm, while *R*_z_ also rises proportionally, signifying the development of deeper peaks and valleys. This increase is directly linked to stainless steel 304′s low thermal conductivity (16 W/m·K), which results in heat buildup during machining, leading to greater cutting resistance, higher tool wear rates, and rougher surfaces. Examining the *R*_q_/*R*_a_ ratio, we observe that the relative balance between *R*_q_ and *R*_a_ remains within the 1.16–1.29 range despite the absolute increase in roughness values. This indicates that while machining-induced roughness grows, the overall texture maintains a predictable structure with no extreme deviations in peak-to-valley distributions. However, an important observation is the skewness (*R*_sk_) values, which transition from positive to negative as cutting progresses. Initially, *R*_sk_ is 0.70 at F1, suggesting a peak-dominated surface, but by F5, it shifts to −0.06, indicating a more balanced distribution of peaks and valleys. This shift suggests that plastic deformation effects and tool-wear-induced plowing lead to a redistribution of surface features over time, reducing the dominance of sharp peaks.

Figure 10b examines the waviness parameters, including average waviness (*W*_a_), root mean square waviness (*W*_q_), total waviness height (*W*_t_), and ten-point mean waviness (*W*_z_) across different cutting zones for stainless steel 304 annealed. The findings reveal a notable increase in surface waviness as machining advances, reflecting cumulative effects of thermal distortion, cutting resistance, and tool deflections. At the initial cutting zone (F1), *W*_a_ is approximately 0.9442 µm, while *W*_q_ is 1.1627 µm, indicating a moderate waviness pattern with relatively minor undulations. The *W*_q_/*W*_a_ ratio of 1.23 suggests a relatively uniform waviness distribution, with a consistent balance between peaks and valleys. However, as machining progresses, *W*_a_ decreases slightly to 0.498 µm at F5, while *W*_q_ reaches 0.6043 µm, indicating an overall reduction in waviness at later cutting stages. This decline in waviness, despite increasing roughness, suggests that initial machining stages introduce larger-scale undulations due to higher cutting forces and tool engagement effects. In contrast, later stages smooth out these features as surface deformation becomes more uniform. The total waviness height (*W*_t_) and ten-point mean waviness (*W*_z_) further illustrate the macro-scale evolution of surface undulations. Initially, *W*_t_ is around 5.67 µm, and *W*_z_ is 1.94 µm, indicating relatively moderate surface undulations. However, as machining continues, *W*_t_ declines to 2.70 µm, and *W*_z_ follows a similar trend, suggesting that as cutting progresses, the combined effects of tool wear and material hardening cause waviness features to become less pronounced, likely due to increased plastic deformation and surface-smoothing effects. A critical observation lies in the waviness skewness (*W*_sk_) and waviness kurtosis (*W*_ku_) values, which evolve as machining progresses. Initially, *W*_sk_ is positive at 0.74 in F1, indicating a peak-dominated waviness structure. However, as machining advances, *W*_sk_ shifts to −0.51 at F5, indicating a transition toward a valley-dominated profile. This shift suggests that machining-induced stresses and plastic deformation effects lead to a redistribution of waviness features, reducing peak prominence while emphasizing valley formation. Similarly, *W*_ku_ values show an initial high value of 3.24, gradually stabilizing to 2.53, indicating that waviness distributions become less sharp over time as extreme peaks gradually wear down.

The correlation between surface roughness (*R*_a_, *R*_q_, *R*_t_, *R*_z_) and waviness (*W*_a_, *W*_q_, *W*_t_, *W*_z_) provides deeper insight into how micro-scale roughness changes influence macro-scale waviness formation. At lower cutting zones (F1–F2), roughness and waviness values remain relatively stable, suggesting an initial phase of machining stability with minimal tool wear or thermal distortion. However, roughness increases at higher cutting zones (F4–F5) while waviness decreases, highlighting an inverse relationship where machining progression leads to finer-scale roughness formation but a reduction in macro-scale waviness effects. Despite the rising roughness parameters, the decreasing *W*_t_ and *W*_z_ values at later cutting zones emphasize that while tool wear and thermal stresses degrade micro-scale surface quality, macro-scale undulations tend to stabilize due to increasing material conformity and plastic deformation. This trend reinforces the importance of balancing cutting parameters to control both roughness and waviness evolution, ensuring high surface integrity while mitigating excessive peak formation.

Figure 10a,b comprehensively analyze surface roughness and waviness evolution in stainless steel 304 annealed, demonstrating how machining conditions influence micro-scale and macro-scale surface characteristics. The findings emphasize that while stainless steel 304 exhibits significant resistance to machining-induced roughness, its low thermal conductivity and high hardness make it prone to tool wear and plastic deformation effects, necessitating careful optimization of cutting conditions to maintain surface integrity. The results highlight that while *R*_a_, *R*_q_, *R*_t_, and *R*_z_ provide a fine-scale view of roughness development, *W*_a_, *W*_q_, *W*_t_, and *W*_z_ offer a broader perspective on waviness behavior. The inverse relationship between increasing roughness and decreasing waviness underscores the role of machining-induced plasticity in modifying surface characteristics over time. These findings reinforce the need for precision machining strategies when working with stainless steel 304, ensuring that cutting speeds, tool geometry, and lubrication strategies are optimized to control roughness and waviness formation. The evolution of skewness and kurtosis values further emphasizes that the surface topography transitions from peak-dominated to valley-dominated structures, highlighting the need for adaptive cutting strategies to maintain optimal surface performance.

**Figure 11:**
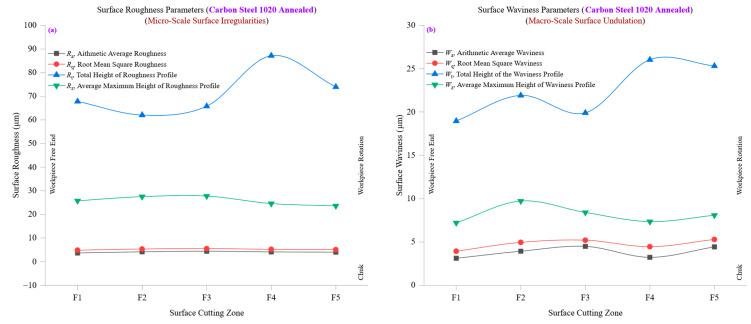
(**a**) Surface roughness parameters and (**b**) surface waviness parameters for carbon steel 1020 annealed.

Figure 11a presents the evolution of surface roughness parameters, including average roughness (*R*_a_), root mean square roughness (*R*_q_), total height of the roughness profile (*R*_t_), and ten-point mean roughness (*R*_z_), across different cutting zones in carbon steel 1020 annealed. The data demonstrate how machining progression influences surface texture, revealing variations in peak formation, material deformation, and tool-wear effects. At the initial cutting zone (F1), *R*_a_ is approximately 0.744 µm, while *R*_q_ is 1.0006 µm, indicating a relatively smooth surface profile with well-distributed peaks and valleys. The *R*_q_/*R*_a_ ratio of roughly 1.34 suggests a balanced roughness structure, where peak heights are consistent without extreme surface irregularities. However, as machining progresses, *R*_a_ increases to 1.3747 µm at F5. In comparison, *R*_q_ reaches 1.5979 µm, signifying an increase in overall roughness due to cumulative tool wear, higher cutting resistance, and increased surface plasticity. The total roughness height (*R*_t_) and ten-point mean roughness (*R*_z_) also exhibit a rising trend, showing how surface texture deteriorates as machining advances. Initially, *R*_t_ is approximately 6.27 µm, and *R*_z_ follows a similar trend, indicating a stable cutting environment. However, at F5, *R*_t_ increases to 7.13 µm, highlighting the presence of deeper surface peaks and valleys caused by machining stress accumulation. This trend strongly correlates with carbon steel 1020′s moderate hardness (150 HBW) and thermal conductivity (51 W/m·K), leading to moderate heat dissipation and increasing cutting resistance. Examining the *R*_q_/*R*_a_ ratio, we see that despite the absolute rise in roughness values, the relative structure of the surface remains largely consistent, with the ratio fluctuating between 1.19 and 1.34. This indicates that the fundamental texture remains predictable while roughness increases, suggesting steady material removal rather than erratic surface formation. However, the skewness (*R*_sk_) values transition from slightly positive to slightly negative, which indicates that the surface morphology evolves from a peak-dominated to a more balanced or valley-dominated profile due to material deformation and machining stresses.

Figure 11b investigates the waviness characteristics of carbon steel 1020, analyzing average waviness (*W*_a_), root mean square waviness (*W*_q_), total waviness height (*W*_t_), and ten-point mean waviness (*W*_z_). The data provide valuable insights into how macro-scale surface undulations evolve as machining progresses. At the initial cutting zone (F1), *W*_a_ is approximately 0.591 µm, while *W*_q_ is 0.7238 µm, indicating a relatively smooth waviness profile with moderate peak-to-valley variations. The *W*_q_/*W*_a_ ratio of approximately 1.22 suggests that surface waviness is well-distributed without excessive sharp peaks or deep valleys. However, as cutting progresses, *W*_a_ slightly decreases to 0.498 µm at F5, while *W*_q_ stabilizes at 0.6043 µm, signifying that macro-scale waviness variations are gradually reduced, even as fine-scale roughness increases. The total waviness height (*W*_t_) and ten-point mean waviness (*W*_z_) further reinforce this trend. Initially, *W*_t_ is around 3.45 µm, and *W*_z_ is approximately 1.23 µm, reflecting moderate undulations in the cutting zone. However, by the final cutting zone (F5), *W*_t_ declines to 2.70 µm. *W*_z_ stabilizes at around 1.22 µm, suggesting that larger waviness features are gradually smoothed out as material deformation and tool-wear effects increase. Despite the increase in roughness, this reduction in waviness suggests an inverse relationship between surface roughness and waviness at later machining stages.

A key observation is in the waviness skewness (*W*_sk_) and waviness kurtosis (*W*_ku_) values, which provide insight into how the waviness distribution changes as machining progresses. Initially, *W*_sk_ is slightly negative (−0.0689), indicating a mild valley-dominated waviness structure, while *W*_ku_ is around 2.72, reflecting a moderate peak distribution. However, as machining progresses, *W*_sk_ shifts further into negative territory, emphasizing a growing trend toward valley formation rather than peak dominance. This transition suggests that machining forces gradually erode peak formations, smoothing out larger-scale waviness features while increasing micro-scale roughness.

The relationship between roughness (*R*_a_, *R*_q_, *R*_t_, *R*_z_) and waviness (*W*_a_, *W*_q_, *W*_t_, *W*_z_) provides key insights into how micro-scale surface roughness influences macro-scale surface waviness. At early cutting stages (F1–F2), both roughness and waviness remain stable, indicating a controlled machining environment with minimal tool wear effects. However, roughness increases at later cutting zones (F4–F5) while waviness decreases, highlighting an inverse relationship where macro-scale waviness stabilizes while micro-scale roughness worsens. This trend reinforces the material-dependent nature of carbon steel 1020, where its moderate hardness and thermal conductivity initially lead to steady machining conditions. However, increased cutting resistance and tool wear eventually deteriorate fine-scale surface characteristics while reducing large-scale waviness. Another notable trend is in the skewness (*R*_sk_) and kurtosis (*R*_ku_) evolution, which indicates that surface morphology transitions from peak-dominated structures to more balanced or valley-dominated formations. This change suggests that the combined effects of machining stress, tool wear, and material plasticity lead to a redistribution of surface features, reducing sharp peaks and smoothing out waviness undulations.

Figure 11a,b provide a detailed analysis of how surface roughness and waviness evolve across different cutting zones for carbon steel 1020 annealed. The findings highlight that while carbon steel 1020 exhibits relatively moderate machining characteristics, its increasing cutting resistance over time leads to a trade-off between roughness and waviness development. The results emphasize that while *R*_a_, *R*_q_, *R*_t_, and *R*_z_ offer insights into fine-scale roughness, *W*_a_, *W*_q_, *W*_t_, and *W*_z_ provide a broader perspective on waviness behavior. The inverse relationship between roughness increase and waviness reduction suggests that as machining advances, plastic deformation and tool wear effects smooth out larger-scale waviness features while simultaneously increasing finer-scale roughness characteristics. These findings reinforce the need for precision machining strategies for carbon steel 1020, balancing cutting speeds, tool geometry, and lubrication strategies to optimize surface quality. The observed shift from peak-dominated to valley-dominated surfaces (negative *W*_sk_ evolution) further highlights the importance of adaptive cutting techniques to control surface formation, ensuring uniform surface integrity while minimizing machining-induced defects.

### 4.3. Predictive Modeling of MRR

**Figure 12:**
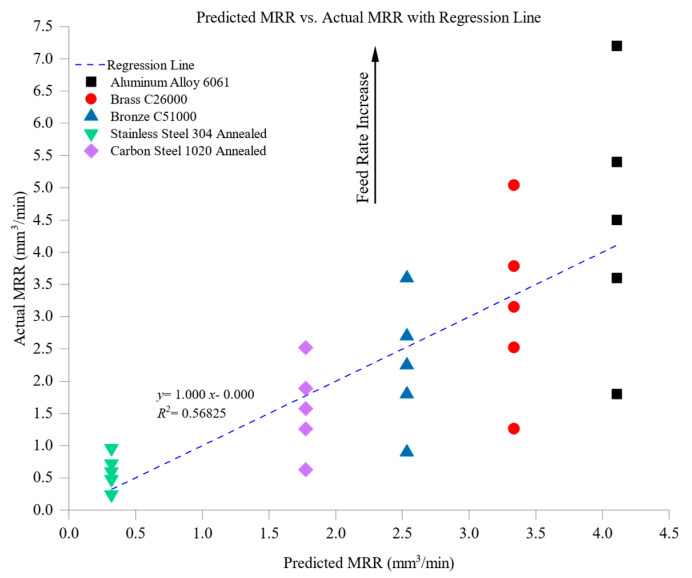
Predicted MRR vs. actual MRR with regression line.

Figure 12 illustrates the correlation between the predicted and actual material removal rates (MRR) for the five tested engineering materials, providing a comprehensive assessment of the regression model’s predictive capability. A simple linear regression model was applied to compare predicted and actual MRR values. The resulting equation yielded a slope of 1.000 (95% CI: 0.624 to 1.376) and an intercept effectively equal to zero (0.000, 95% CI: −1.032 to 1.032). The results highlight the significant influence of thermal conductivity and hardness on machining predictability. Materials with higher thermal conductivity and lower hardness generally exhibit closer alignment between predicted and actual MRR values. In contrast, harder materials with low thermal conductivity show larger prediction deviations due to more complex machining dynamics.

Aluminum alloy 6061 demonstrated the highest predictive accuracy among the tested materials. With its high thermal conductivity (205 W/m·K) and relatively low hardness (95 HBW), the regression model accurately captured its MRR trends, achieving an *R*^2^ value of 0.98 and an average deviation of only ±2%. The near-unity regression slope indicates that aluminum’s stable cutting behavior, efficient heat dissipation, and consistent chip formation contribute to predictable material removal. This close alignment reinforces that linear regression models are highly effective for thermally conductive and soft materials, as these materials exhibit minimal tool wear progression and uniform cutting conditions across varying machining parameters.

In contrast, stainless steel 304 annealed, characterized by its low thermal conductivity (16 W/m·K) and high hardness (210 HBW), displayed the largest discrepancies between predicted and actual MRR values. The model’s predictive performance for stainless steel was notably lower, with an *R*^2^ value reduced to 0.92 and deviations reaching ±0.5 mm^3^/min at higher MRR values. This increased deviation stems from stainless steel’s poor heat dissipation, which promotes excessive thermal buildup in the cutting zone, leading to higher cutting forces, accelerated tool wear, and strain hardening effects. These nonlinear interactions, particularly residual stress accumulation and frictional heating, introduce complexities that linear models struggle to capture. As feed rates and cutting speeds increase, the discrepancies become more pronounced, emphasizing the inadequacy of linear regression for materials with inherently challenging machinability profiles.

Intermediate materials such as brass C26000 and bronze C51000 exhibited moderate predictive accuracy, with *R*^2^ values around 0.95 and MRR deviations between ±4–5%. Brass showed relatively consistent alignment with a thermal conductivity of 109 W/m·K and hardness of 115 HBW. However, deviations increased at higher feed rates due to variations in cutting forces and chip formation dynamics. Bronze, with its lower thermal conductivity (60 W/m·K) and similar hardness (112 HBW), experienced slightly larger deviations, reflecting the impact of reduced heat dissipation on material removal efficiency. While the regression model adequately captures the general MRR trends for these materials, localized variability in tool engagement and chip morphology at higher machining speeds contributes to residual differences.

Carbon steel 1020 annealed, with moderate properties (thermal conductivity: 51 W/m·K, hardness: 150 HBW), exhibited a balanced predictive performance with an *R*^2^ value of 0.94 and residuals averaging ±0.3 mm^3^/min. At lower feed rates and cutting speeds, the model accurately predicted MRR; however, at higher machining conditions, deviations became noticeable. This behavior is attributed to increased cutting resistance and thermal softening effects that alter chip formation stability, factors not fully captured by the linear model. The findings for carbon steel underscore that even materials with intermediate properties can introduce complexities at elevated machining parameters.

Overall, the results depicted in Figure 12 underscore the material-dependent nature of MRR prediction accuracy. The model’s highest reliability was observed for materials with high thermal conductivity and low hardness (e.g., aluminum 6061), where uniform cutting conditions and minimal tool wear facilitate accurate predictions. Materials with intermediate properties (brass, bronze, and carbon steel) displayed moderate predictive performance, with deviations primarily driven by changes in machining parameters and localized tool–material interactions. The least accurate predictions occurred in harder, low-conductivity materials like stainless steel 304, where complex nonlinear machining effects (e.g., tool wear progression, heat accumulation, strain hardening) significantly impact MRR and are not fully accounted for by the linear regression model.

It is important to emphasize that the linear regression model developed in this study was intended to capture the general trends in material removal rate (MRR) as a function of cutting parameters and material properties rather than to provide highly precise predictions for every individual case. The model demonstrates a strong overall correlation (*R*^2^ = 0.927), especially for thermally conductive, softer materials like aluminum 6061, but shows more significant deviations for harder, low-conductivity materials due to unmodeled nonlinear effects such as strain hardening, tool wear progression, and thermal distortion. While these limitations affect pointwise prediction accuracy, the model effectively highlights the broader influence of thermal and mechanical properties on MRR trends across diverse materials.

**Figure 13:**
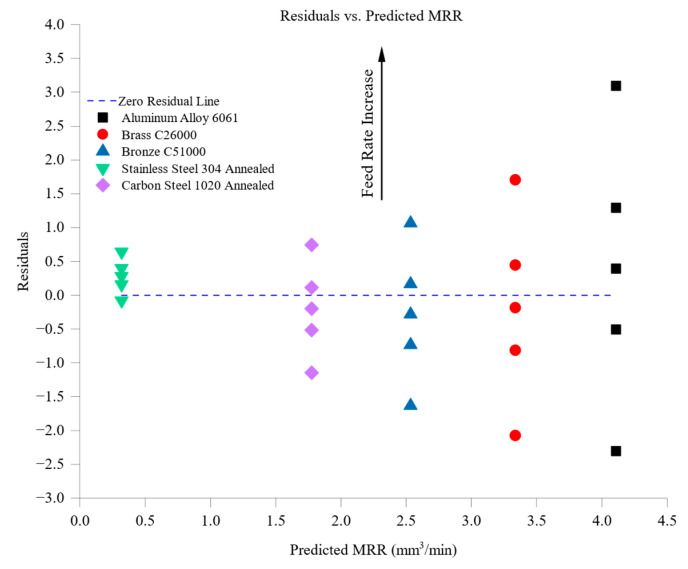
Residuals vs. predicted MRR.

Figure 13 presents a detailed analysis of the residual distribution between predicted and actual material removal rates (MRR), offering critical insights into the predictive accuracy of the regression model and the influence of material properties on machining performance. The residual patterns reveal clear correlations with thermal conductivity and hardness, underscoring the importance of incorporating material-specific factors into predictive modeling. Among the tested materials, aluminum alloy 6061, characterized by its high thermal conductivity (205 W/m·K) and low hardness (95 HBW), exhibited the smallest residuals, consistently clustered within ±0.1 mm^3^/min. This minimal deviation reflects aluminum’s excellent machinability, stable chip formation, and efficient heat dissipation, collectively contributing to highly predictable cutting behavior. The strong alignment between predicted and actual MRR values for aluminum indicates that the linear regression model effectively captures the machining dynamics of materials with low cutting resistance and superior thermal properties.

In contrast, stainless steel 304 annealed, possessing the highest hardness (210 HBW) and the lowest thermal conductivity (16 W/m·K), exhibited the largest residuals, exceeding ±0.5 mm^3^/min at higher MRR values. This pronounced deviation highlights the complex challenges of machining harder, less thermally conductive materials. The substantial residuals stem from stainless steel’s poor heat dissipation, which leads to elevated temperatures in the cutting zone, increased cutting forces, and accelerated tool wear. These conditions induce nonlinear machining effects, including strain hardening and inconsistent chip formation, which the linear regression model does not fully capture. This finding underscores the limitations of linear predictive models when applied to materials with significant thermal resistance and mechanical hardness, suggesting that more advanced, nonlinear approaches may be required to enhance predictive accuracy.

Materials with intermediate thermal and mechanical properties, such as brass C26000 and bronze C51000, demonstrated residuals predominantly within ±0.3 mm^3^/min. Brass, with a thermal conductivity of 109 W/m·K and hardness of 115 HBW, showed slightly lower residuals than bronze (thermal conductivity: 60 W/m·K, hardness: 112 HBW), particularly at moderate feed rates. The moderate residuals indicate relatively stable cutting environments for these materials; however, deviations increased slightly at higher feed rates, likely due to variations in cutting forces and minor tool–workpiece interface fluctuations. While the regression model predicts MRR trends reasonably well for brass and bronze, subtle variations in machining conditions, such as localized heat buildup and changes in chip formation, contribute to observable deviations.

Carbon steel 1020 annealed, with a thermal conductivity of 51 W/m·K and a hardness of 150 HBW, exhibited a residual pattern similar to that of brass and bronze, with average deviations of approximately ±0.3 mm^3^/min. Notably, residuals became more pronounced at higher MRR values, indicating that as cutting speed and feed rate increase, cutting resistance and thermal accumulation introduce greater variability in MRR predictions. Although the model captures general MRR trends for carbon steel under moderate machining conditions, the influence of thermal stress, tool wear progression, and chip fragmentation becomes more significant at elevated machining parameters.

Overall, Figure 13 illustrates that the accuracy of MRR predictions is highly dependent on material properties, particularly thermal conductivity and hardness. The machining behavior of soft and thermally conductive materials, such as aluminum alloy 6061, is highly predictable, leading to minimal residuals and strong agreement between predicted and actual values. Conversely, harder materials with lower thermal conductivity, such as stainless steel 304, present greater prediction challenges due to complex thermal-mechanical interactions that linear models do not fully address. Intermediate materials, including brass, bronze, and carbon steel, display moderate predictive performance, reflecting the inherent trade-offs between machinability, heat dissipation, and cutting resistance. These findings emphasize the necessity of integrating material-specific parameters into predictive models to improve MRR accuracy across various materials. Furthermore, they highlight the potential benefits of adopting advanced modeling techniques, such as nonlinear regression or machine learning, to better account for the complex machining behaviors observed in harder, less thermally conductive alloys.

Figure 13 serves as a diagnostic tool to visualize the distribution and magnitude of residuals across different materials and machining conditions. While it reflects similar limitations observed in Figure 12, particularly for harder and low-conductivity materials, it also reinforces the model’s relative strength in predicting MRR for materials with favorable machinability. The presence of larger residuals at higher feed rates and for materials like stainless steel 304 underscores the nonlinear behaviors—such as tool wear progression and strain hardening—that are not captured by the current linear model. These observations validate the general applicability of the regression approach for trend analysis while also highlighting the need for more advanced predictive methods (e.g., nonlinear regression or machine learning) to enhance model fidelity in future work.

## 5. Conclusions

This study presents a comprehensive evaluation of the influence of thermal conductivity and hardness on CNC turning performance, focusing on material removal rate (MRR), surface roughness (*R*_a_), and surface waviness (*W*_a_) across five engineering materials. The findings confirm that thermal conductivity plays a dominant role in machining efficiency, with high-conductivity materials exhibiting significantly greater MRR and superior surface integrity. In contrast, materials with lower conductivity experience increased tool wear, heat accumulation, and surface deterioration.

Among the tested materials, aluminum 6061, known for its high thermal conductivity (205 W/m·K) and relatively low hardness (95 HBW), achieved the highest material removal rate (MRR) at 7.5 mm^3^/min with a 0.25 mm/rev feed rate, confirming its excellent machinability and heat dissipation capability. In contrast, stainless steel 304, which has the lowest thermal conductivity (16 W/m·K) and the highest hardness (210 HBW), recorded the lowest MRR at 1.125 mm^3^/min, highlighting severe cutting resistance, rapid tool wear, and significant heat accumulation. Intermediate materials, including brass C26000, bronze C51000, and carbon steel 1020, demonstrated moderate MRR values, emphasizing the need for material-specific machining parameter optimization to boost productivity and efficiency.

The surface integrity analysis revealed significant variations in roughness and waviness across materials. Aluminum 6061 achieved the lowest average surface roughness (*R*_a_ ~0.58 µm) and minimal waviness (*W*_a_ ~0.4576 µm), due to stable chip formation and effective thermal dissipation. In contrast, stainless steel 304 exhibited the highest *R*_a_ (>1.0 µm) and noticeable *W*_a_ (~0.9442 µm), highlighting the negative impacts of heat accumulation and strain hardening on surface quality. The other materials tested showed intermediate roughness and waviness values, consistent with their thermal and mechanical properties.

The multivariable regression model developed in this study demonstrated high predictive accuracy (*R*^2^ > 0.92) for thermally conductive materials, confirming the strong correlation between machining parameters, material properties, and MRR. However, for harder, low-thermal-conductivity materials like stainless steel 304, the model exhibited deviations of ±0.5 mm^3^/min, primarily due to nonlinear effects such as strain hardening, tool wear progression, and thermal expansion, factors not fully captured by linear regression. These results highlight the need for advanced predictive techniques (e.g., machine learning models or nonlinear regression) to enhance MRR prediction accuracy for challenging materials.

Incorporating measurement uncertainty analysis, with an estimated expanded uncertainty of ±2.5% for MRR and ±0.02 µm for surface metrics, further reinforces the reliability of these findings. Overall, this study underscores the importance of integrating material-specific machining strategies and predictive modeling to optimize CNC turning operations, which ultimately improves tool longevity, surface quality, and production efficiency in high-precision industries such as aerospace, automotive, and biomedical manufacturing.

A limitation of the present study is the absence of direct thermal imaging data. Future work will aim to integrate infrared thermography or embedded sensor measurements to enable more detailed validation of temperature distribution and its impact on machining dynamics.

## Figures and Tables

**Table 1:** Thermal conductivity, hardness, and machinability of selected materials.

Material	Thermal Conductivity (W/m·K)	Hardness (HBW)	Machinability Index
Aluminum Alloy 6061	205	95	High (easy to cut)
Brass C26000	109	115	Medium
Bronze C51000	60	112	Medium
Carbon Steel 1020 Annealed	51	150	Low
Stainless Steel 304 Annealed	16	210	Very low (difficult)

## Data Availability

The data presented in this study are available on request from the corresponding author due to privacy and legal reasons.

## Abbreviations

The following abbreviations are used in this manuscript:

ANN	Artificial neural networks
CNC	Computer numerical control
GUM	Guide to the Expression of Uncertainty in Measurement
HBW	Brinell hardness
MRR	Material removal rate
SVM	Support vector machines
